# Mixed Ammonium-Nitrate Nutrition Regulates Enzymes, Gene Expression, and Metabolic Pathways to Improve Nitrogen Uptake, Partitioning, and Utilization Efficiency in Rice

**DOI:** 10.3390/plants14040611

**Published:** 2025-02-18

**Authors:** Xianting Fan, Chusheng Lu, Zaid Khan, Zhiming Li, Songpo Duan, Hong Shen, Youqiang Fu

**Affiliations:** 1 College of Natural Resources and Environment, South China Agricultural University, Guangzhou 510642, China; fxt2443612196@163.com (X.F.); lcs2450345066@163.com (C.L.); zaidkhan@scau.edu.cn (Z.K.); gxulzm2017@163.com (Z.L.); songpo.duan@hotmail.com (S.D.); 2 Rice Research Institute, Guangdong Academy of Agricultural Sciences, Guangzhou 510640, China; 3 Guangdong Key Laboratory of Rice Science and Technology, Guangzhou 510640, China; 4 Key Laboratory of Genetics and Breeding of High-Quality Rice in Southern China (Co-Construction by Ministry and Province), Ministry of Agriculture and Rural Affairs, Guangzhou 510640, China

**Keywords:** rice, ammonium-nitrate mixed nutrition, nitrogen metabolism enzymes, transcriptomics, root morphology

## Abstract

Ammonium and nitrate nitrogen are the two main forms of inorganic nitrogen (N) available to crops. However, it is not clear how mixtures of ammonium and nitrate N affect N uptake and partitioning in major rice cultivars in southern China. This study investigated the effects of different ammonium nitrogen and nitrate nitrogen mixture treatments (100:0, 75:25, 50:50, 25:75, and 0:100) on the growth, photosynthetic characteristics, nitrogen uptake, gene expression, and yield of different rice cultivars (Mei Xiang Zhan NO. 2: MXZ2; Nan Jing Xiang Zhan: NJXZ). Rice root biomass, tiller number, and yield were increased by 69.5%, 42.5%, and 46.8%, respectively, in the 75:25 ammonium-nitrate mixed treatment compared to the 100:0 ammonium-nitrate mixed treatment. The nitrogen content in rice roots, stems, leaves, and grains increased by 69.5%, 64.0%, 65.5%, and 17.5%, respectively. In addition, compared with MXZ2, NJXZ had a greater proportion of N allocated to leaves and grains. Analysis of root enzyme activities revealed that the 75:25 ammonium-nitrate mixed nutrient treatment increased rice root glutamine synthetase activity by an average of 35.0% and glutamate synthetase activity by an average of 52.0%. Transcriptome analysis revealed that the 75:25 mixed ammonium-nitrate nutrient treatment upregulated the expression of genes related to the nitrogen metabolism transporter pathway. Weighted correlation network analysis revealed that some differentially expressed genes (*HISX* and *RPAB5*) regulated the activities of nitrogen-metabolizing enzymes in rice and some (*SAT2*, *CYSKP*, *SYIM*, *CHI1*, and *XIP1*) modulated amino acid synthesis; greater expression of these genes was detected in the 75:25 ammonium-nitrate mixed nutrient treatment. The expression characteristics of the above genes were further confirmed by RT‒qPCR. Interestingly, the expression levels of the above genes were significantly correlated with the glutamate synthase activity, photosynthetic rate, and root volume. It is noteworthy that increasing the expression of the aforementioned genes coupled with nitrogen uptake was observed in the three main rice cultivars. These results suggest that the 75:25 ammonium-nitrate mixture may have increased nitrogen-metabolizing enzyme activities and promoted nitrogen uptake through the upregulated expression of nitrogen metabolism-related genes, thereby increasing tiller number and improving rice yield.

## 1. Introduction

Rice (*Oryza sativa* L) is a crucial food crop that provides sustenance for nearly half of the world’s population [1]. To meet the basic needs of a growing global population, ensuring high quality and high yield for rice is strategically important. Rice plants display disparate responses to and preferences for ammonium and nitrate nitrogen. These responses manifest at both the physiological and morphological levels, with notable distinctions in the uptake and utilization of these nitrogen sources, as well as in physiological regulatory processes [2]. In soil environments with good aeration conditions, rice plants take up nitrate nitrogen as the main form of nitrogen, and under flooded conditions, rice roots mainly take up ammonium nitrogen as the main form of nitrogen [3,4]. However, rice cultivation techniques such as sunbathing, wet and dry alternation, and aerobic cultivation in the middle stage of production, as well as the rice root system’s own secretion of oxygen, can convert ammonium nitrogen to nitrate nitrogen; thus, the rice root system exists in a nutrient environment where ammonium nitrogen and nitrate nitrogen are mixed. Therefore, because of ammonium-nitrate mixing in the nutrient environment of rice, it is vital to study the uptake mechanism and regulation strategy for ammonium nitrogen and nitrate nitrogen in rice to optimize nutrient management and improve the efficiency of nutrient utilization, which is vital for promoting sustainable agricultural development.

Nitrogen is a key nutrient in plant growth and is the main component of many important organic compounds in plants. For example, nitrogen is an essential component of nucleic acids, amino acids, proteins, chlorophyll, enzymes, etc. [5]. Nitrogen fertilization strongly affects plant growth, development, yield, and quality [6,7]. Therefore, it is important to study the efficient application of nitrogen fertilizers to improve the efficiency of nitrogen use in rice and increase yield. Except for some plants that derive nitrogen from nitrogen-fixing microorganisms that fix atmospheric N_2_ as an absorbable source of nitrogen, most plants absorb and utilize both organic and inorganic nitrogen from the soil. The main forms of nitrogen absorbed by plants are ammonium nitrogen (NH_4_^+^) and nitrate nitrogen (NO_3_^−^), followed by urea and amino acids. Ammonium nitrogen and nitrate nitrogen are being increasingly used in agricultural production, and high crop yields are often achieved by applying large amounts of nitrogen fertilizer. However, the overapplication of nitrogen fertilizers and low plant nitrogen use efficiency have resulted in large amounts of residual nitrogen fertilizers in soil, which has led to serious effects on the agroecological environment [8,9]. Under current policies, seeking to improve the efficiency of plant nitrogen use and maintain high quality and yield while reducing nitrogen fertilizer application is crucial at the agricultural, environmental, and economic levels [10]. However, increasing nitrogen use efficiency through the provision of appropriate ammonium-nitrate nitrogen mixtures for nutrition is a complex challenge and involves physiological and molecular mechanisms ranging from nitrogen uptake and assimilation to translocation and utilization [11]. Therefore, it is crucial to improve the nitrogen utilization efficiency of crops by studying the physiological and molecular mechanisms of different ammonium-nitrate nitrogen mixtures used for nutritional purposes [12].

At the same time, mixed ammonium-nitrate nutrition can promote the growth and development of the rice root system and improve the efficiency of nutrient absorption and utilization by rice. The rice root system absorbs ammonium nitrogen and nitrate nitrogen from the soil and assimilates them into amino acids, mainly through the glutamine synthetase/glutamate synthetase-mediated 2-oxoglutarate aminotransferase (GS/GOGAT) cycle, which increases the amino acid content of the rice root system and promotes its upward translocation to the aboveground part of the plant via the xylem, thereby increasing the nitrogen utilization efficiency of rice [13]. In addition, ammonium-nitrate nitrogen mixtures can upregulate the expression of related genes, increase nitrogen-metabolizing enzyme activities, and promote nitrogen uptake by rice, thereby increasing rice yield [14]. The molecular mechanism of the response of rice to nitrogen nutrition can be studied through transcriptome analysis to determine the possible nitrogen response strategies, which will help improve the efficiency of nitrogen utilization in rice and increase rice yield [15]. Current research focuses on the uptake and utilization efficiency and physiological mechanisms of ammonium-nitrate nutrient blends applied to plants, as well as the environmental adaptation and abiotic stress effects of ammonium-nitrate nutrient blends on plant growth and development [16,17,18]. Therefore, we speculate that the mixed ammonium-nitrate nutrition might regulate enzymes, gene expression, and metabolic pathways to improve nitrogen uptake, partitioning, and utilization efficiency in rice. However, the molecular mechanisms and expression of key genes involved in the process of nitrogen conversion and utilization in rice under different ammonium-nitrate nitrogen mixed nutrient conditions have rarely been reported. This study examines the physiological processes of rice plants treated with various ammonium-nitrate nutrient mixtures. Molecular biological techniques, such as transcriptomics, are utilized to reveal metabolic pathways related to the nitrogen response of rice roots under mixed ammonium-nitrate nutrient conditions. Additionally, this study investigated the mechanism affecting the process of nitrogen uptake and translocation in rice. The findings of this study will serve as a scientific reference for the efficient application of nitrogen fertilizers in rice.

## 2. Results

### 2.1. Effects of Various Ammonium-Nitrate Nutrient Combinations on Rice Plant Development

**Figure 1:**
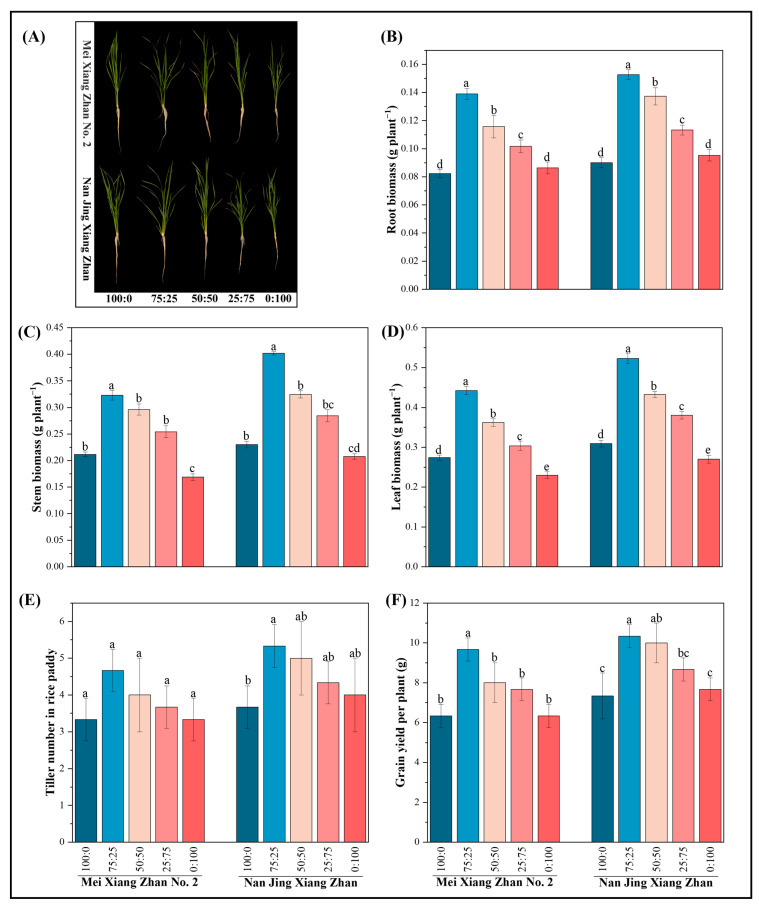
Effect of different ammonium-nitrate nutrient mixtures on rice phenology, biomass, and yield. The figure shows (**A**) rice growth phenotype, (**B**) root biomass, (**C**) stem biomass, (**D**) leaf biomass, (**E**) tiller number per plant, and (**F**) grain yield per plant. According to Fisher’s protected LSD test (*p* < 0.05), different lowercase letters indicate significant differences between treatments of the same cultivar. 100:0 ammonium-nitrate mixed nutrient, 75:25 ammonium-nitrate mixed nutrient, 50:50 ammonium-nitrate mixed nutrient, 25:75 ammonium-nitrate mixed nutrient, 0:100 ammonium-nitrate mixed nutrient.

Rice roots, stems, and leaves showed significant differences under different ammonium-nitrate mixed nutrient conditions (Figure 1A). The biomass of rice roots, stems, and leaves showed an increasing and then decreasing trend as the proportion of ammonium nitrogen in the ammonium-nitrate nutrient mixture decreased, regardless of whether the MXZ2 or NJXZ rice cultivar was used (Figure 1B–D). The biomass of roots, stalks, and leaves increased by 69%, 53%, and 62%, respectively, in the MXZ2 rice cultivar and 70%, 75%, and 69%, respectively, in the NJXZ rice cultivar when the ammonium-nitrate ratio was 75:25 compared to the treatment with an ammonium-nitrate ratio of 100:0. The number of tillers and yield of the MXZ2 rice cultivar increased by 40% and 52%, respectively, with the 75:25 ammonium-nitrate mixture compared with the 100:0 ammonium-nitrate mixture, but there was no significant difference in the number of tillers of the MXZ2 cultivar among the ammonium-nitrate mixture treatments. The number of tillers and yield of the NJXZ rice cultivar increased significantly, by 45% and 45%, respectively, compared to those in the 100:0 ammonium-nitrate mixed nutrient treatment (Figure 1E,F). The biomass of roots, stems, and leaves, the number of tillers, and the yield per plant of NJXZ were significantly greater than those of MXZ2 in the corresponding treatments.

### 2.2. The Impact of Various Ammonium-Nitrate Nutrient Combinations on the Morphology of Rice Roots

**Figure 2:**
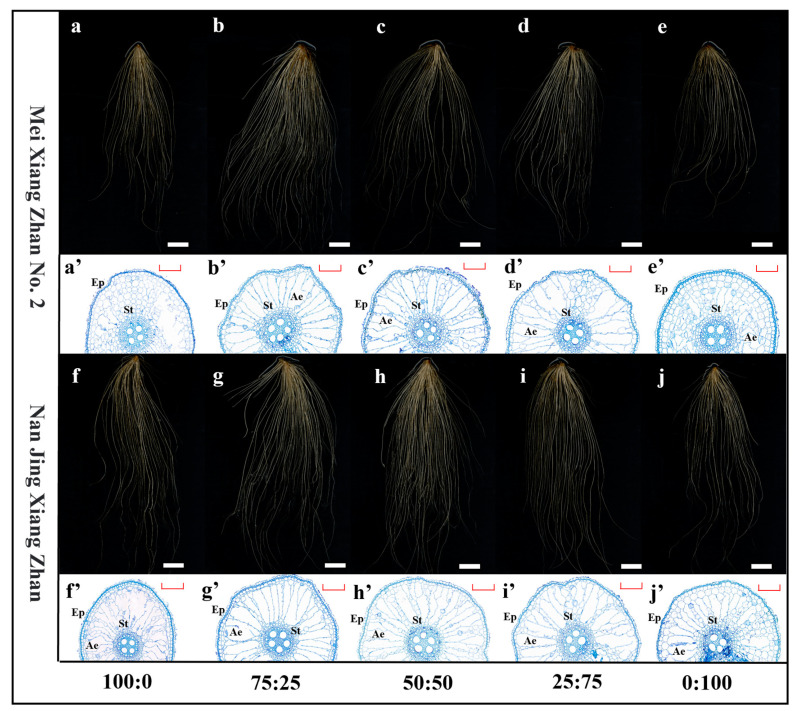
Rice root growth under different ammonium–nitrate nutrient mixtures. (**a**–**j**) represent root RGB images of different rice cultivars under different ammonium-nitrate nutrient mixtures, and (**a’**–**j’**) represent root cross sections after toluidine blue staining. St, stele. Ae, aerenchyma. Ep, epidermis. Scale bars = 4 cm (**a**–**e**), 5 cm (**f**–**j**), and 100 µm (**a’**–**j’**). According to Fisher’s protected LSD test *p* < 0.05), different lowercase letters indicate significant differences between treatments of the same cultivar.

The total root length, total root surface area, average root diameter, total root volume, and number of root tips of the rice cultivars first increased significantly and then decreased as the proportion of ammonium nitrogen in the ammonium-nitrate mixture decreased in the MXZ2 and NJXZ rice cultivars. Compared with those in the 100:0 treatment, the total root length, total root surface area, mean root diameter, total root volume, and number of rice root tips significantly increased in the 75:25 ammonium-nitrate mixed nutrient treatment. The increases were 84%, 48%, 22%, 84%, and 96%, respectively, for the MXZ2 rice cultivar and 91%, 97%, 21%, 72%, and 236%, respectively, for the NJXZ rice cultivar (Table 1). Compared with those under the 100:0 ammonium-nitrate mixed nutrient treatment, the root systems of both the MXZ2 and NJXZ rice cultivars showed an increase in xylem growth and vigorous development of aeration tissues when the ammonium-nitrate mixed nutrient ratio was 75:25 (Figure 2). The results showed that the 75:25 ammonium-nitrate mixture promoted the growth of the rice root system and provided an advantage for the upward delivery of the ammonium-nitrate mixed nutrients in rice compared with the 100:0 ammonium-nitrate mixed nutrient treatment. Compared with the 100:0 ammonium-nitrate mixed nutrient treatment, the 75:25 ammonium-nitrate mixed nutrient treatment increased xylem growth and the development of aeration tissues and decreased cell deformation in the rice root system.

### 2.3. Effect of Nitrogen Form on the Activity of Enzymes Involved in Nitrogen Synthesis in Rice

**Figure 3:**
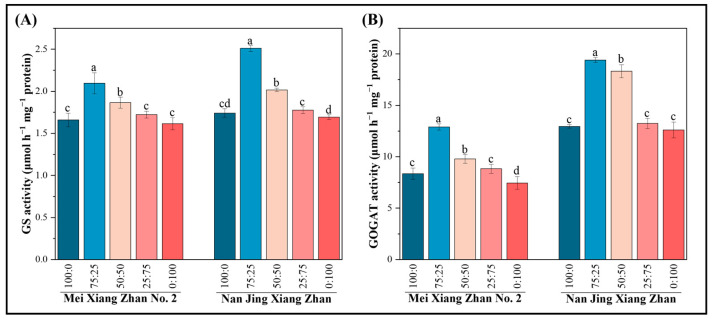
Effects of different ammonium-nitrate nutrient mixtures on the activities of key enzymes involved in nitrogen metabolism in rice. The figure shows the (**A**) glutamine synthetase activity and (**B**) glutamate synthase activity. According to Fisher’s protected LSD test (*p* < 0.05), different lowercase letters indicate significant differences between treatments of the same cultivar.

The findings showed that the various nutritional conditions affected the activity of important enzymes involved in nitrogen metabolism in rice roots under different ammonium–nitrate combinations (Figure 3). The glutamate synthase and glutamine synthetase activities in the rice roots tended to first increase and then decrease when the amount of ammonium nitrogen in the ammonium-nitrate nutrient mixture decreased (Figure 3). When treated with the 75:25 ammonium-nitrate mixture rather than the 100:0 ammonium-nitrate mixture, the glutamine synthetase activity and glutamate synthetase activity in rice roots increased by 26–44% and 54–50%, respectively, in the MXZ2 and NJXZ cultivars. The activities of nitrogen-metabolizing enzymes in the root system of NJXZ were significantly greater than those in the root system of MXZ2, and NJXZ showed significant upregulation of glutamate synthetase activity under ammonium-nitrate ratios of 75:25 and 50:50.

### 2.4. Effects of Various Ammonium-Nitrate Nutrient Combinations on the Photosynthetic Pigments and Processes in Rice Leaves

**Figure 4:**
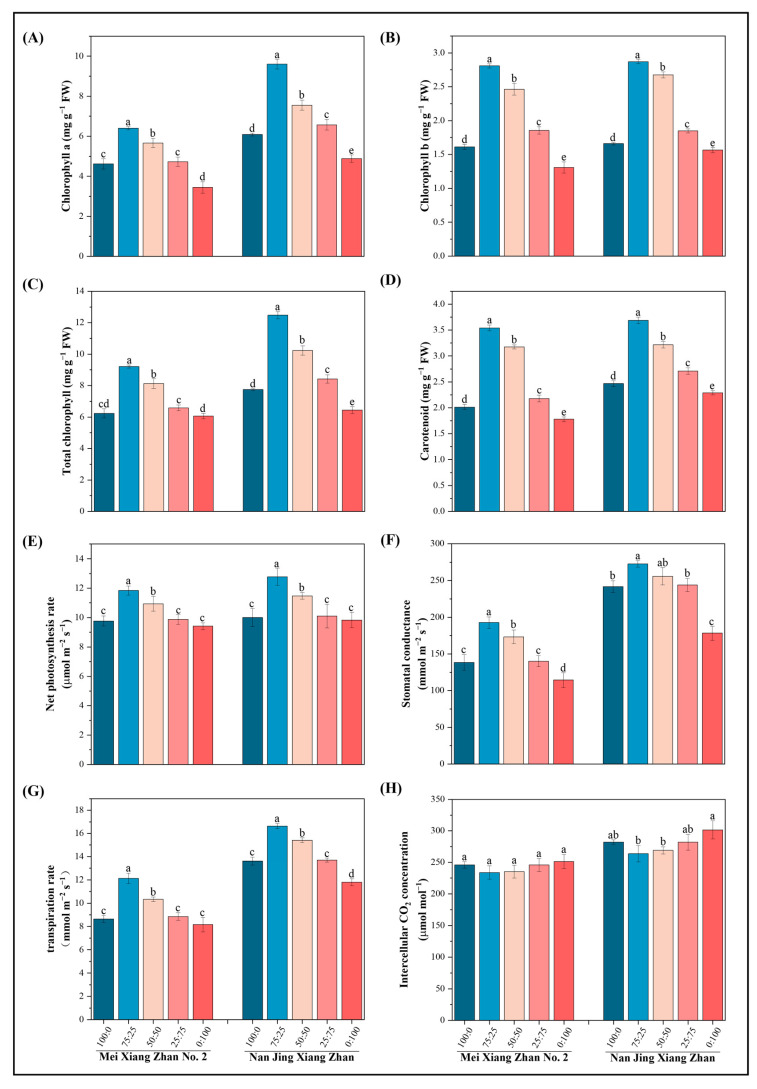
Effect of different ammonium-nitrate mixtures on the photosynthetic characteristics of rice leaves. The figure shows the (**A**) chlorophyll a, (**B**) chlorophyll b, (**C**) carotenoid, (**D**) and total chlorophyll levels, (**E**) net photosynthetic rate, (**F**) stomatal conductance, (**G**) transpiration rate, and (**H**) intercellular CO_2_ concentration. According to Fisher’s protected LSD test (*p* < 0.05), different lowercase letters indicate significant differences between treatments of the same cultivar.

The photosynthetic pigments and rates in the rice leaves significantly decreased with increasing nitrate nitrogen content in the ammonium-nitrate mixtures in MXZ2 and NJXZ (Figure 4). The chlorophyll a, chlorophyll b, total chlorophyll, and carotenoid contents increased by 39%, 73%, 48%, and 81%, respectively, in the MXZ2 rice cultivar and by 84%, 37%, 70%, and 37%, respectively, in the NJXZ rice cultivar compared with those in the 100:0 ammonium-nitrate mixed nutrient treatment (Figure 4A–D). Compared with the 100:0 ammonium-nitrate mixed nutrient treatment, the 75:25 ammonium-nitrate mixed nutrient treatment increased the transpiration rate, photosynthetic rate, and stomatal conductance by 40%, 21%, and 39%, respectively, in the MXZ2 rice cultivar and 22%, 28%, and 13%, respectively, in the NJXZ rice cultivar (Figure 4E–G). The intercellular CO_2_ concentration was reduced by 5% in the MXZ2 rice cultivar and 7% in the NJXZ rice cultivar when the ammonium-nitrate mixed nutrient ratio was 75:25 compared to when the ammonium-nitrate mixed nutrient ratio was 100:0 (Figure 4H). The results showed that the photosynthetic capacity of the NJXZ rice cultivar was significantly greater than that of the MXZ2 cultivar under the same treatment. The photosynthetic characteristics of the leaves of the two rice cultivars were more strongly affected by the 75:25 ammonium-nitrate mixture than by the 100:0 ammonium-nitrate mixture.

### 2.5. Nitrogen Content of Rice Tissues Under Various Ammonium-Nitrate Nutritional Combinations

**Figure 5:**
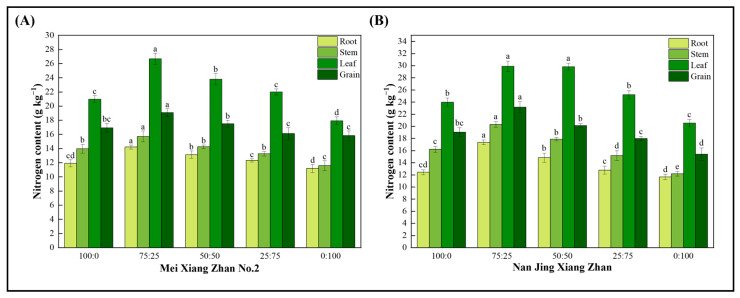
Effect of different ammonium-nitrate nutrient mixtures on the nitrogen content of rice tissues. (**A**) rice cultivar Mei Xiang Zhan No.2; (**B**) rice cultivar Nan Jing Xiang Zhan. Common lowercase letters indicate significant differences between treatments according to Fisher’s protected LSD test (*p* < 0.05).

The nitrogen content of rice roots, stems, and leaves tended to increase and subsequently decrease when the amount of ammonium nitrogen in the ammonium-nitrate mixed nutrient system decreased, regardless of the rice cultivar (MXZ2 or NJXZ) (Figure 5). The nitrogen content of the rice roots, stems, leaves and seeds was significantly greater under treatment with the 75:25 ammonium-nitrate mixture than with the other ammonium-nitrate mixtures (100:0, 50:50, 25:75, and 0:100). Compared with the 100:0 ammonium-nitrate mixed fertilizer, the 75:25 ammonium-nitrate mixed fertilizer increased the nitrogen content by 19%, 12%, 27%, and 13% in the rice roots, stems, leaves and seeds of MXZ2, respectively, and by 40%, 25%, 25%, and 22% in the rice roots, stems, leaves and seeds of NJXZ, respectively. The nitrogen uptake capacity of the NJXZ rice cultivar was significantly greater than that of the MXZ2 cultivar. In addition, both rice cultivars accumulated N mainly in the leaves. These results indicated that the 75:25 ammonium-nitrate mixture significantly increased the N content of rice tissues compared to the other ammonium-nitrate mixtures (100:0, 50:50, 25:75, and 0:100) and may have promoted nitrogen uptake and utilization in rice.

### 2.6. Gene Expression in Rice Roots Under Different Ammonium–Nitrate Mixtures

**Figure 6:**
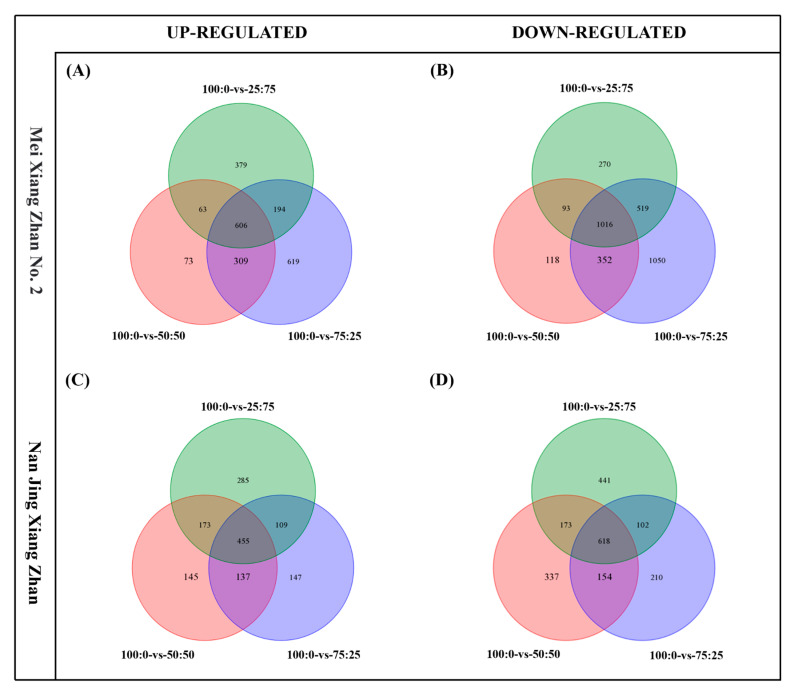
Venn diagrams showing significant up- and downregulated genes in rice roots under different ammonium-nitrate mixed nutrient conditions. As indicated by (**A**), there is up-regulated gene expression between different treatments of the MXZ2 rice cultivar. As indicated by (**B**), there is down-regulated gene expression between different treatments of the MXZ2 rice cultivar. As indicated by (**C**), there is up-regulated gene expression between different treatments of the NJXZ rice cultivar. As indicated by (**D**), there is down-regulated gene expression between different treatments of the NJXZ rice cultivar.

To reveal the molecular mechanism of nitrogen transport, two rice cultivars with significant differences in nitrogen efficiency were cultured in ammonium-nitrate mixed nutrient treatments (100:0, 75:25, 50:50, and 25:75) for 30 days and then subjected to transcriptomic analysis. The results showed that in the root system of MXZ2, 1728, 1051, and 1242 upregulated DEGs and 2937, 1579, and 1898 downregulated DEGs were found in the comparisons of 100:0 vs. 75:25, 100:0 vs. 50:50, and 100:0 vs. 25:75, respectively. Similarly, in the root system of NJXZ, 848, 910, and 1022 upregulated DEGs and 1084, 1282, and 1334 downregulated DEGs were found in the comparisons of 100:0 vs. 75:25, 100:0 vs. 50:50, and 100:0 vs. 25:75, respectively. According to the Venn diagram, there were gains of 606 DEGs and losses of 1016 DEGs in MXZ2, gains of 455 DEGs and losses of 618 DEGs in NJXZ among the 100:0, 75:25, 50:50, and 25:75 treatments, respectively (Figure 6).

### 2.7. Rice Root RNA Sequencing to Examine the Response to Nitrogen Supply

The results of GO and KEGG enrichment studies were used to gain a deeper understanding of the role played by DEGs and their connection to pertinent biological processes. Twenty GO keywords were used to classify the DEGs associated with various ammonium-nitrate nutrient mixes into three categories: cellular composition, molecular activities, and biological processes (Figure S1). A majority of the DEGs in these categories, as shown in Figure S1, were mainly enriched in membrane, ribonucleotide binding, transferase activity, protein kinase activity, transferring glycosyl groups, cellular protein modification process, transmembrane transporter activity, and transport. Cultivars with MXZ2 showed gene enrichment in transferase activity and membrane. Genes in the cellular component category, which were mainly enriched in membrane, were significantly more abundant in rice cultivars with NJXZ than those with MXZ2 (Figure S1B–F).

Significantly enriched DEGs involved in secondary metabolite biosynthesis, metabolic pathways, plant‒pathogen interactions, phenylpropanoid biosynthesis, and glutathione metabolism were found by KEGG enrichment analysis (Figure S2). The number of DEGs related to the biosynthesis of secondary metabolites, metabolic pathways, plant‒pathogen interactions, phenylpropanoid biosynthesis, and glutathione metabolism significantly increased in MXZ2 under 100:0 vs. 75:25 (Figure S2E). Compared with MXZ2, NJXZ showed significant enrichment of metabolic pathways under 100:0 vs. 75:25 (Figure S2E,F). More significant enrichment of DEGs involved in metabolism was observed in rice with NJXZ than in rice with MXZ2, and the nitrogen conversion and utilization processes associated with the enriched DEGs were superior to those in the MXZ2 cultivars.

### 2.8. Identification and Comparison of Key Genes

To identify genes associated with nitrogen uptake and utilization in rice, we performed WGCNA for all genes. Gene modules were classified based on clustering relationships between genes, and genes with similar expression patterns were classified into the same module, resulting in the identification of 21 modules (Figure S3A). Correlation analysis was performed using module eigenvalues with data for specific traits and phenotypes to identify the black modules, green modules, and purple modules that were most relevant to the traits and phenotypes (Figure S3B). The key genes in the black, purple, and green modules showed significant correlations with glutamate synthase, root volume, and transpiration rate (correlation > 0.8). GO enrichment analysis of the genes in these black, green, and purple modules (Figure S3C–E) revealed that the key genes were mainly enriched in nitrogen compound metabolism, cellular nitrogen compound metabolism process, and organic nitrogen compound metabolism process, which play important roles in nitrogen uptake and transport in rice.

**Figure 7:**
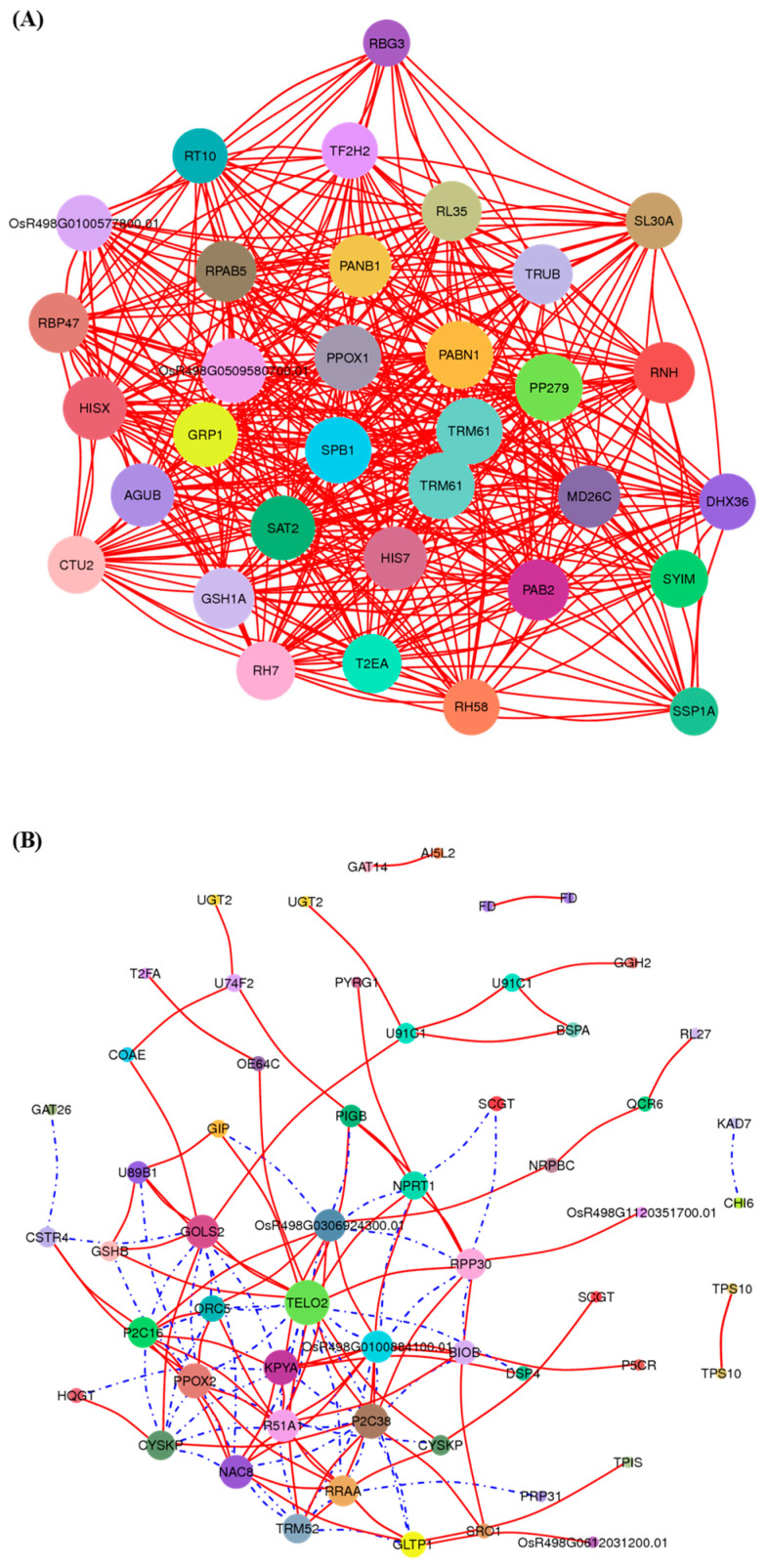

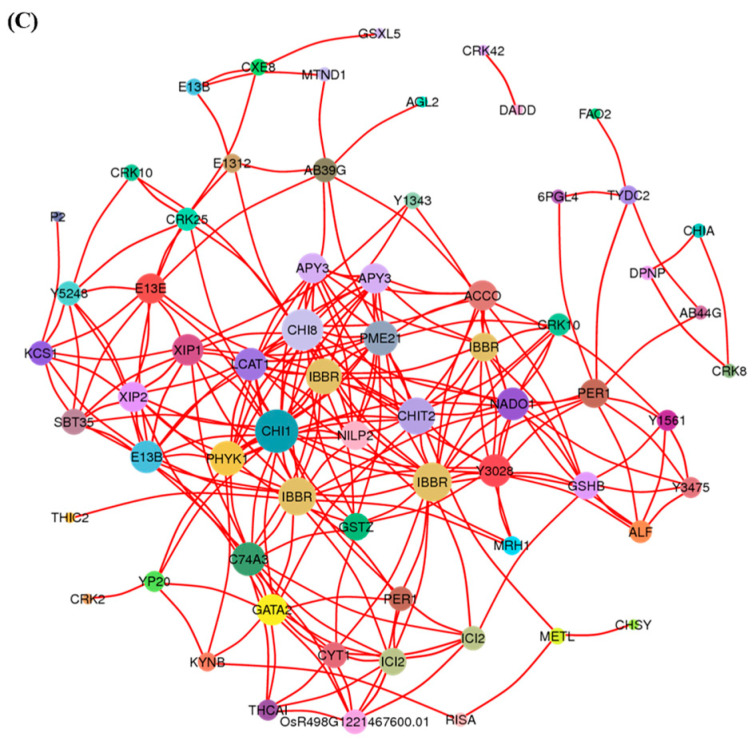
(**A**) visualization of the first 30 genes in the black module, (**B**) visualization of the first 30 genes in the green module, and (**C**) visualization of the first 30 genes in the purple module.

Genes related to nitrogen uptake and transport utilization were selected by GO enrichment analysis and imported into Cytoscape software to construct gene coexpression network maps. The core genes that were pivotal in the network of regulatory relationships of the black module were *HISX*, *RPAB5*, *SAT2*, and *SYIM* (Figure 7A). These genes play important roles mainly in the processes of histidine biosynthesis, ribonucleic acid polymerase, amino acid biosynthesis, cysteine and methionine metabolism, and aminoacyl biosynthesis. The core gene that was pivotal in the network of regulatory relationships of the green module was *CYSKP* (Figure 7B); the gene is involved in amino acid biosynthesis and cysteine and methionine metabolism. The core genes that were pivotal in the network of regulatory relationships of the purple module were *CHI1* and *XIP1* (Figure 7C). These genes are mainly associated with and play important roles in amino and nucleotide sugar metabolism, arginine and proline metabolism, tryptophan metabolism, and phenylalanine metabolism.

### 2.9. Differential Gene Expression in Rice Roots Under Different Ammonium-Nitrate Nutrient Mixture Conditions

**Figure 8:**
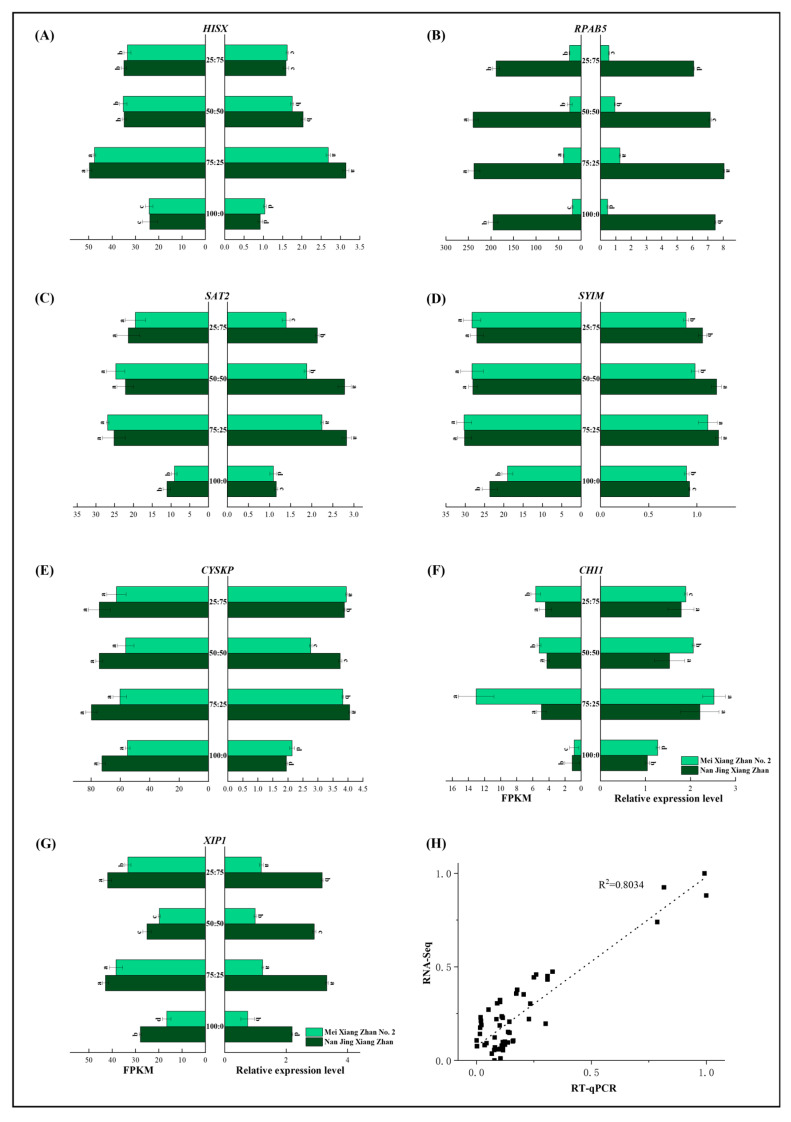
Correlation of the normalized fold change with RNA-seq results after RT‒qPCR validation of the seven key hubs. (**A**) RT‒qPCR analysis of *HISX*, (**B**) RT‒qPCR analysis of *RPAB5*, (**C**) RT‒qPCR analysis of *SAT2*, (**D**) RT‒qPCR analysis of *SYIM*, (**E**) RT‒qPCR analysis of *CYSKP*, (**F**) RT‒qPCR analysis of *CHI1*, (**G**) RT‒qPCR analysis of *XIP1*, and (**H**) correlation analysis of normalized fold changes from RT‒qPCR data and RNA‒seq data. Different lowercase letters indicate significant differences between treatments at *p* < 0.05.

To validate the RNA-seq data, we performed RT‒qPCR analysis of seven key hub genes identified by weighted gene coexpression network analysis in rice (Figure 8A–G). The FPKM values and relative expression levels (RT‒qPCR data) of the seven selected genes showed the same trend among treatments, and the linear regression coefficient (R^2^) between the RT‒qPCR and RNA-seq results was 0.8034 (Figure 8H). This finding confirms the reliability of the transcriptome (RNA-seq) data obtained in previous experiments. We also found that the expression of these key genes was significantly greater under the 75:25 condition than under the 100:0 condition (Figure 8A–G). Except for *CHI1*, the expression of genes in NJXZ was significantly greater than that in MXZ2 (Figure 8F).

### 2.10. Expression of Differential Genes in the Root System of Different Rice Cultivars Under Mixed Ammonium-Nitrogen Nutrient Conditions

The expression of the RPAB5, CYSKP, and XIP1 genes was investigated in the root systems of three major cultivars grown in southern China (Table 2). The results showed that *RPAB5*, *CYSKP*, and *XIP1* were increased by 46.5%, 24.6%, and 58.4%, respectively, in root system of rice WY308 under 75:25 ammonium-nitrogen mixture treatment compared with 100:0 ammonium-nitrogen mixture nutrition; *CYSKP*, *XIP1* genes were up-regulated by 18.8% and 29.2% in the root system of SY9516; The genes *RPAB5*, *CYSKP*, and *XIP1* were up-regulated and expressed by 19.6%, 85.7%, and 86.9%, respectively, in the WFY615 root system. In addition, the nitrogen content of the three rice roots increased by 6.9%, 25.2%, and 9.2% under the 75:25 ammonium-nitrate mixed nutrient conditions. This suggests that *RPAB5*, *CYSKP*, and *XIP1* genes are involved in nitrogen uptake and transport. The GO Analytics database annotation results further support the above view that *RPAB5*, *CYSKP*, and *XIP1* are involved in nitrogen uptake and translocation (Figure S3).

## 3. Discussion

The rice root system is the main organ for nutrient uptake, and vigorous root growth can promote nutrient uptake and utilization [19]. Nitrogen is a crucial element in the growth process of rice. The supply of ammonium and nitrate nitrogen promotes the growth and development of the rice root system [20]. Rice root systems exhibit different responses to ammonium and nitrate nitrogen. A localized supply of nitrate promoted primary and secondary lateral root elongation in Arabidopsis, whereas a supply of ammonium nitrogen promoted secondary and tertiary lateral root density [21]. Nitrate nitrogen regulates endogenous growth hormone uptake in root cells, stimulates lateral root development, and promotes lateral root growth. However, the assimilation of nitrate by plants requires large amounts of energy, and nitrate tends to migrate with soil moisture runoff in a short period, leading to a decrease in nitrogen use efficiency [22]. Boschiero et al. [23] demonstrated that a 75:25 ammonium-nitrate mixture significantly promoted root growth in sugarcane and maize [24]. Consistent with previous studies, the present study revealed that ammonium-nitrate mixed nutrient treatment resulted in consistent rice root growth (Figure 2 and Table 1). The above results showed that the suitable ammonium-nitrate mixed nutrient treatment promoted the full branching of the rice root system, increased the nutrient uptake area, and improved nutrient uptake and utilization by the root system.

Different modes of nitrogen supply affect plant photosynthesis [25]. Nitrogen is essential for regulating chlorophyll synthesis in crop leaves, and there is a strong positive correlation between a plant’s nitrogen content and its chlorophyll content [26,27]. Nitrogen metabolism provides energy for photosynthesis in rice leaves, and the greater photosynthetic capacity of leaves stimulates the conversion of light energy and CO_2_ into chemical energy, which ultimately results in the production of more photosynthetic products, thereby increasing crop biomass and yield [28]. *Malus hupehensis* seedlings exhibited the highest chlorophyll (a, b, and a + b) content and the lowest leaf respiration rate when the ammonium-nitrate ratio was 50:50 [29]. The chlorophyll a, chlorophyll b, and carotenoid levels in oilseed rape leaves peaked under a 7.5:7.5 treatment, whereas the leaf net photosynthesis (Pn), stomatal conductance (Gs), and transpiration rate (Tr) increased with increasing ammonium nitrogen in the nutrient solution [30]. In the present study, the chlorophyll a, chlorophyll b, total chlorophyll, and carotenoid contents, transpiration rate, photosynthesis rate, and stomatal conductance were significantly greater in the leaves of both rice species at an ammonium-nitrate ratio of 75:25, and the reason for this result may be related to cultivar-specific characteristics (Figure 4).

The ammonium nitrogen taken up by the rice root system is assimilated into amino acids by rice via the glutamate synthase (GOGAT)/glutamine synthase (GS) pathway, and nitrate taken up is reduced to NH_4_^+^, which is subsequently assimilated into amino acids by the rice plant via the GOGAT/GS pathway [31]. It was found that GS and GOGAT had greater activity when an ammonium-nitrate nitrogen mixture was used [32,33]. Moreover, suitable ammonium-nitrate nitrogen blends promote crop growth and increase the number of tillers in rice [23,34]. The dry matter content of chili peppers in the presence of a 50:50 ammonium-nitrate mixture peaked at 120 days [35]. Crops such as oilseed rape, sugarcane, and rice have a preference for NH_4_^+^, but with an appropriate increase in the supply of NO_3_^−^, the crops showed the best growth response with ammonium-nitrate mixed nutrient treatments, with the highest level of growth achieved at an ammonium-nitrate ratio of 75:25 [23,36]. The results of this study showed that an ammonium-nitrate ratio of 75:25 significantly promoted root growth in both rice cultivars, increased root nitrogen-metabolizing enzyme activity (Figure 3), promoted root nitrogen uptake, facilitated aboveground growth, and increased yield. The possible reason for this is that ammonium is a source of nitrogen that can be directly absorbed and utilized by plants, and rice plants have a preference for ammonium nitrogen, as nitrate nitrogen uptake requires more energy [37]. Suitable ammonium-nitrate nutrient mixtures can increase N partitioning to seeds and decrease N partitioning to stems and roots, thereby improving nitrogen utilization efficiency [38]. In the present study, the 75:25 ammonium-nitrate mixture promoted more N accumulation in the leaves and grains of the two rice cultivars, probably because of the characteristics of ammonium and nitrate N uptake in rice (Figure 5). Therefore, in the nitrogen management strategies of rice, we suggest supplying a certain proportion of nitrate nitrogen fertilizer in rice production, which can reduce nitrogen input, decrease nitrogen loss to the environment, improve nitrogen use efficiency and enhance agricultural sustainability to a certain extent.

Transcriptomics is a method for studying gene expression at the RNA level; this method quantifies the level of gene expression with high precision and is an important tool for studying cell phenotype and function [39]. The application of different ammonium-nitrate N ratios significantly affected gene expression [40]. Yu’s study revealed that the *NAR21* and *NAR22* genes can regulate the expression of genes encoding nitrate transporter proteins, promote nitrate accumulation, and control the development of primary and lateral roots through NO_3_^−^ [41]. In this study, via transcriptomic analysis, we determined the expression levels of nitrogen response genes in the rice root system at the tillering stage and detected upregulated expression of the nitrate nitrogen transport genes *NAR21* and *NAR22* in the rice root system of MXZ2 at an ammonium-nitrate mixed nutrient ratio of 75:25 and upregulated expression of *NAR21* and *NAR22* in the rice root system of NJXZ at an ammonium-nitrate mixed nutrient ratio of 50:50. This suggests that mixed ammonium-nitrate nutrition is important for improving nitrogen utilization efficiency in different rice cultivars and that the specific ammonium-nitrate mixing ratios that are most suitable may vary among rice cultivars. Comparative transcriptome analysis revealed that the significantly differentially expressed genes were enriched mainly in pathways related to nitrogen synthesis, and the expression of these genes was significantly greater under the ammonium-nitrate ratio of 75:25 than under the other ammonium-nitrate ratios. Therefore, after co-analysis of the physiological traits of rice plants with differentially expressed genes revealed that the differentially expressed genes were significantly correlated with glutamate synthase activity, photosynthetic rate, and root volume, WGCNA was used to further identify hub genes responsive to nitrogen uptake and translocation in the rice root system. WGCNA revealed seven pivotal genes affecting nitrogen use efficiency in rice. We found that seven (*HISX*, *RPAB5*, *SAT2*, *SYIM*, *CYSKP, CHI1*, and *XIP1*) were associated with nitrogen synthesis and highly expressed under an ammonium-nitrate mixed nutrient ratio of 75:25 (Figure 7). The *HISX* gene is responsible for regulating GS and GOGAT activity during histidine biosynthesis [42]. The *RPAB5* gene encodes an RNA polymerase that is involved in the production of glutamine synthetase [43]. Both the *HISX* and *RPAB5* genes regulate nitrogen utilization in rice by affecting glutamate synthetase and glutamine synthetase. The *SAT2* and *CYSKP* genes are responsible for amino acid biosynthesis, which is a key process in the uptake and utilization of nitrogen from the soil in rice and regulates the efficiency of nitrogen uptake and utilization in plants [44]. The *SYIM* gene encodes an aminoacyl-tRNA, which is a substrate for translation and is key in determining how the genetic code is translated to amino acids [45]. The *CHI1* and *XIP1* genes are responsible for aminosugar and nucleotide sugar metabolism, the conversion of nucleotide sugars to amino acids through a series of enzyme-catalyzed reactions [46]. Therefore, proper regulation of the ratio of ammonium nitrogen to nitrate nitrogen can affect the expression of genes related to nitrogen metabolism in rice, thus affecting the uptake and utilization of nitrogen in rice and promoting rice growth. This result also confirmed that the key genes were strongly correlated with glutamate synthase activity, photosynthetic rate, and root volume (Figure S3B), which provides ideas and a theoretical basis for the identification of nitrogen-responsive genes in rice.

## 4. Materials and Methods

### 4.1. Test Material

The rice cultivars Nanjing Xiang Zhan (NJXZ) and Mei Xiang Zhan 2 (MXZ2), along with WY308, SY9516, and WFY615, which are extensively cultivated in South China, were utilized as test materials in this study. The experiment was conducted in a controlled greenhouse of the Department of Plant Nutrition, College of Resources and Environment, South China Agricultural University. The temperature was set at 25–28 °C for 10 h during the day/14 h at night, and the artificial light intensity was activated to maintain between 15,000 and 30,000 lux (lx) when the natural light is below 15,000 lx.

### 4.2. Experimental Design

The selected seeds with full grains were sterilized with H_2_O_2_ for 20 min, washed with pure water, and placed in Petri dishes for germination. Seedlings were then incubated in ¼ full nutrient solution for 7 days, and at the three-leaf heart stage, the seedlings were conducted five treatments (i.e., 100% NH_4_^+^-N (100:0), 75% NH_4_^+^-N + 25% NO_3_^−^-N (75:25), 50% NH_4_^+^-N + 50% NO_3_^−^-N (50:50), 25% NH_4_^+^-N+75% NO_3_^−^-N (25:75), and 100% NO_3_^−^-N (0:100)) with a total nitrogen concentration of 2.86 mmol L^−1^ for 90 days. The pH of the nutrient solution was adjusted to 5.5 daily, and the nutrient solution was changed every three days. The NH_4_^+^-N donor was NH_4_Cl, and the NO_3_^−^-N donor was NaNO_3_. Dicyandiamide (7.0 μmol L^−1^), a nitrification inhibitor, was added to all treatments to eliminate microbial effects. The other components of the nutrient solution, except for the different nitrogen forms, were amended with the nutrient solution according to the International Rice Research Institute (IRRI) as follows. The following macronutrients were added (mmol L^−1^): KH_2_PO_4_·2H_2_O, 0.32; K_2_SO_4_, 0.84; CaCl_2_, 1.0; MgSO_4_·7H_2_O, 1.7. The following micronutrients were added (μmol L^−1^): MnCl_2_·4H_2_O, 9.1; H_2_MoO_4_, 0.52; H_3_BO_3_, 18; ZnSO_4_·7H_2_O, 0.15; CuSO_4_·5H_2_O, 0.1616; and Fe (III)-EDTA, 150.

### 4.3. Sampling and Measurement

#### 4.3.1. Measurement of Physiological Properties

After a 90-day ammonium-nitrate nitrogen mixture treatment, rice rhizomes and leaves were harvested separately, and one portion was washed with distilled water, frozen immediately in liquid nitrogen, and stored in a −80 °C freezer for biochemical analysis. The other portion of the rice rhizomes and leaves was weighed and placed in an oven at 105 °C for 30 min and then adjusted to 80 °C to dryness until a constant weight was reached, after which the dry matter mass of each part was determined [47].

#### 4.3.2. Root Conformation and Physiological Characterization

The root morphology and physiological characteristics were studied after 90 days of nitrogen treatment. Roots were washed with deionized water, and images were obtained using an Expression 12000XL scanner (Epson America, Inc., Los Alamitos, CA, USA) and analyzed with a root analyzer (WinRHIZO Pro LA2400, Regent, Vancouver, BC, Canada) to determine total root length, root surface area, average root diameter, total root volume, and number of root tips [48].

#### 4.3.3. Rice Root Sectioning and Toluidine Blue Staining

Robust 2 cm-long rice primary roots were removed and immersed in FAA plant fixative (90 mL of 50% ethanol, 5 mL of 100% glacial acetic acid, and 5 mL of 37% methanol) at −20 °C for storage [49]. After staining with toluidine blue, the root structure was observed with Slide Viewer.

#### 4.3.4. Nitrogen-Assimilating Enzymes in the Rice Root System

Rice root samples were collected at the time of harvest and stored at −80 °C. The enzymatic activities of glutamate synthase (GS) and glutamine synthase (GOGAT) were determined by using a commercial kit (Solarbio Kit, Guangzhou, China). The GS and GOGAT activities were determined using a spectrophotometer (Epoch™ 2, Biotek, Winooski, VT, USA) to measure the absorbance at 450 and 540 nm, respectively.

#### 4.3.5. Rice Photosynthesis Data

Using the LI-6400 portable photosynthesis system (LI-COR Inc., Lincoln, NE, USA) from 9 a.m. to 11 a.m. on a sunny day, the photosynthetic rate, stomatal conductance, transpiration rate, and intercellular CO_2_ concentration were measured in the third leaf, with three leaves examined for each treatment [50].

#### 4.3.6. Determination of Chlorophyll a, Chlorophyll b, and Carotenoid Levels in Rice

Chlorophyll was extracted from leaves using a mixture of 45% acetone, 45% ethanol, and 10% distilled water, and the chlorophyll content was determined spectrophotometrically using a UV‒visible spectrophotometer (Epoch™ 2, Biotek, Winooski, VT, USA) to measure the absorbance at 440, 645, and 663 nm [51].

#### 4.3.7. Determination of Nitrogen Accumulation in Plants

After drying, the samples were pulverized, and 0.1 g of each sample was weighed and digested with H_2_SO_4_-H_2_O_2_ to determine the total nitrogen content, after which the nitrogen accumulation was calculated using a continuous-flow analyzer (AutoAnalyer 3, SEAL Analytical, Inc., Norderstedt, Germany) [52].

#### 4.3.8. RNA Extraction and Transcriptome Analysis

Samples were collected for total RNA extraction after 30 days of feeding in nutrient solutions with different ammonium-nitrate mixture treatments (100:0, 75:25, 50:50, and 25:75). Each experiment had three biological replicates. RNA isolation, cDNA library construction, Illumina sequencing, and data processing were performed by Gene Den-ovo Biotechnology Co., Ltd. (Guangzhou, China). The indica rice (R498) genome from the NCBI database was selected as the reference genome (https://www.ncbi.nlm.nih.gov/assembly/GCA_002151415.1/ accessed on 2 May 2023). To estimate the expression levels of the genes, the transcript abundance of each gene was estimated by the reads per kilobase per million (FPKM) values.

#### 4.3.9. Weighted Gene Coexpression Network Analysis (WGCNA)

Coexpression networks were constructed using the WGCNA package in R. After filtering the genes, the gene expression table was imported into R, and the coexpression module was constructed using the default network construction function. Correlation coefficients with samples or sample traits were calculated using module eigenvalues to identify modules related to biology. Intramodule connectivity was calculated for each gene; genes with high connectivity tended to be core genes that may have important functions. The network was visualized using Cytoscape. For the genes in each module, GO and KEGG enrichment analyses were performed to analyze the biological functions of the modules. False discovery rate (FDR) thresholds were obtained by hypergeometric tests, which yielded significantly enriched GO terms and pathways in the FDR < 0.05 module compared to the background.

#### 4.3.10. Root Gene Validation

Fresh rice roots were used to verify the RNA-Seq and WGCNA results, and RT‒qPCR was performed to verify seven key genes and DEGs. RNA extraction was performed after grinding using liquid nitrogen, and reverse transcription and real-time fluorescence quantification were performed as previously described [53]. The primer pairs selected for this study were designed by Primer Premier 6.0, and the details are shown in Table S1. Three independent replicates were employed for each sample, and the relative expression levels were determined using the comparative 2^−ΔΔCt^ method, with *Osactin* as the internal control. Differences were considered statistically significant at *p*< 0.05.

### 4.4. Statistical Analysis

The data are presented as the means with standard errors of three replicates. Statistical analysis was performed using the SPSS 19.0 statistical package and Excel 2016 for Windows. The graphs were generated with Origin 2018 software. In all tables and figures, different lowercase letters indicate significant differences between treatments at *p* < 0.05.

## 5. Conclusions

The 75:25 ammonium-nitrate mixture promoted rice root growth, increased tillering, improved rice leaf photosynthetic parameters, increased nitrogen-metabolizing enzyme activity, and allowed more nitrogen to be distributed in the seed grain. WGCNA with combined physiological indicators revealed seven differentially expressed genes (*HISX*, *RPAB5*, *SAT2*, *SYIM*, *CYSKP*, *CHI1*, and *XIP1*) involved in the regulation of nitrogen assimilation, and these genes were highly expressed in the 75:25 ammonium-nitrate mixed nutrient treatment. This study elucidated the differentially expressed genes involved in nitrogen metabolism in rice roots regulated by ammonium-nitrate mixed nutrition and examined the associated root morphology and physiological mechanism. However, the ionic antagonism and synergism during the uptake of NH_4_^+^ and NO_3_^−^ by the root system still need to be further studied. The results of the study can provide a scientific basis for efficient nitrogen utilization in different rice cultivars.

## Figures and Tables

**Table 1:** Effects of various nutritional ammonium-nitrate combinations on the morphology of rice roots.

Cultivar	Treatment	Length (cm)	Surface Area (cm^2^)	Average Diameter (mm)	Root Volume (cm^3^)	Tips
Mei Xiang Zhan No. 2	100:0	442.48 ± 14.05 c	71.89 ± 0.68 b	0.32 ± 0.02 c	211.55 ± 9.19 cd	647 ± 50.14 d
	75:25	813.91 ± 72.77 a	106.48 ± 19.19 a	0.39 ± 0.02 a	388.21 ± 18.16 a	1271 ± 61.26 a
	50:50	626.08 ± 47.57 b	88.50 ± 5.51 ab	0.37 ± 0.03 ab	285.85 ± 14.05 b	921 ± 55.65 b
	25:75	533.36 ± 40.38 c	77.12 ± 19.67 b	0.36 ± 0.02 ab	255.13 ± 33.38 bc	845 ± 40.95 b
	0:100	491.95 ± 57.14 c	73.14 ± 4.91 b	0.33 ± 0.02 bc	228.46 ± 10.55 cd	758 ± 40.06 c
Nan Jing Xiang Zhan	100:0	653.49 ± 33.58 b	75.27 ± 5.36 b	0.34 ± 0.01 c	324.58 ± 10.00 b	705 ± 8.89 d
	75:25	1247.35 ± 93.89 a	148.32 ± 20.41 a	0.41 ± 0.01 a	559.29 ± 45.86 a	2372 ± 40.70 a
	50:50	772.46 ± 20.60 b	91.49 ± 31.48 b	0.40 ± 0.01 a	361.77 ± 35.43 b	1091 ± 83.64 b
	25:75	702.91 ± 69.02 b	80.83 ± 8.92 b	0.37 ± 0.01 b	359.80 ± 38.56 b	939 ± 38.89 c
	0:100	687.81 ± 28.07 b	77.88 ± 3.52 b	0.35 ± 0.01 c	344.41 ± 18.54 b	860 ± 22.50 c

Note: Data in the table are the mean ± standard error of 3 replicates; different treatments marked with the same small letter indicate non-significant differences (*p* < 0.05).

**Table 2:** Relative expression and nitrogen content of rice root genes under different ammonium-nitrate mix nutrients.

Cultivar	Treatment	*RPAB5*	*CYSKP*	*XIP1*	N Content (g kg^−1^)
WY308	100:0	5.46 ± 0.45 c	2.56 ± 0.38 b	2.19 ± 0.05 b	14.18 ± 0.56 b
	75:25	8.00 ± 0.37 a	3.19 ± 0.05 a	3.47 ± 0.22 a	15.32 ± 0.49 a
	50:50	6.64 ± 0.54 b	2.75 ± 0.15 ab	2.19 ± 0.06 b	13.27 ± 0.34 b
SY9516	100:0	6.68 ± 0.27 ab	1.49 ± 0.17 a	1.30 ± 0.07 a	13.65 ± 0.35 c
	75:25	6.53 ± 0.39 b	1.77 ± 0.32 a	1.68 ± 0.21 a	16.56 ± 0.38 a
	50:50	7.40 ± 0.43 a	1.40 ± 0.18 a	1.38 ± 0.35 a	14.87 ± 0.59 b
WFY615	100:0	6.27 ± 0.27 b	1.26 ± 0.02 c	1.30 ± 0.05 c	16.15 ± 0.26 b
	75:25	7.50 ± 0.36 a	2.34 ± 0.15 a	2.43 ± 0.06 a	17.28 ± 0.41 a
	50:50	6.16 ± 0.03 b	1.83 ± 0.09 b	1.85 ± 0.06 b	16.66 ± 0.09 b

Note: According to Fisher’s protected LSD test (*p* < 0.05), different lowercase letters indicate significant differences between treatments of the same cultivar.

## Data Availability

The original contributions presented in the study are included in the article/Supplementary Material. Further inquiries can be directed to the corresponding author.

## Supplementary Materials

The following supporting information can be downloaded at: https://www.mdpi.com/article/10.3390/plants14040611/s1, Figure S1: Significantly enriched GO terms for differentially expressed genes in rice roots under different ammonium–nitrate mixed nutrient conditions; Figure S2: KEGG enrichment bubble plots for pathway enrichment analysis of rice roots under different ammonium–nitrate mixed nutrient conditions; Figure S3: Weighted gene coexpression network analysis in rice; Table S1: The primer sequences utilized in the RT-qPCR assay are provided below.
