# Supplementary material for: Mixed Ammonium-Nitrate Nutrition Regulates Enzymes, Gene Expression, and Metabolic Pathways to Improve Nitrogen Uptake, Partitioning, and Utilization Efficiency in Rice
Source: Plants (Basel). 2025 Feb 18;14(4):611. doi: 10.3390/plants14040611 (PMC11859190; doi:10.3390/plants14040611)
Supplement: Supplementary file 1 [file plants-14-00611-s001.zip › plants-3384358-supplementary.pdf]

Supplementary Materials

Table S1. The primer sequences utilized in the RT-qPCR assay are provided below.

Gene	Primer	Sequence (5'-3')	PCR Products
Osactin	Forward	CCGCCAGAGAGGAAGTACAG	
	Reverse	AAGCACTTCCTGTGGACGAT	
HISX	Forward	GATTGTTGAAGACGTGCGC	127bp
	Reverse	TGGATCAAGCTCCACATCC	
RPAB5	Forward	CGGGACACCGTTCTTTCC	117bp
	Reverse	CGGGATGATCATCTCCGATG	
SAT2	Forward	GGGCATTGTTGTACCTCAAG	129bp
	Reverse	CCACTGCAAAAACCTCACTG	
SYIM	Forward	ATGTACCGAAAGCTACGAG	148bp
	Reverse	CCTTCATGGAAGTCACAAC	
CYSKP	Forward	GGAATTGGAACAGGTGG	121bp
	Reverse	CAGGTTTTCCACCAGAGA	
CHI1	Forward	TACACCTACGACGCCTT	182bp
	Reverse	TCCTCCTTGAAGCAGTAGC	
XIP1	Forward	GACATCAAGTACTGCCAG	120bp
	Reverse	GTTCCAGAGGTAGTCGT	

(A)

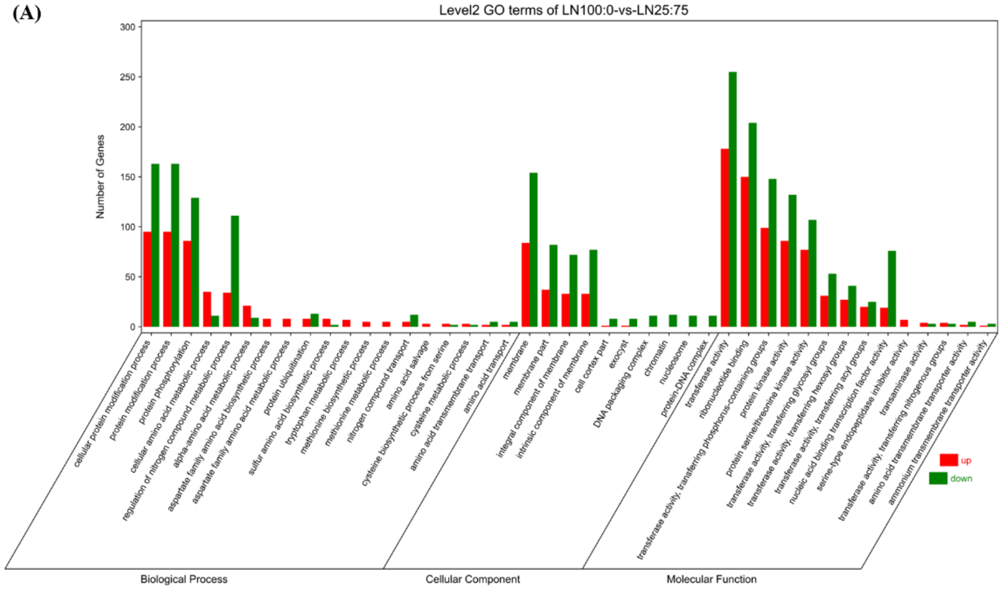
Level2 GO terms of LN100:0-vs-LN25:75
Number of Genes
0
50
100
150
200
250
300
cellular protein modification process
protein modification process
protein phosphorylation
cellular amino acid metabolic process
regulation of nitrogen compound metabolic process
alpha-amino acid metabolic process
aspartate family amino acid biosynthetic process
aspartate family amino acid metabolic process
protein ubiquitination
sulfur amino acid biosynthetic process
tryptophan metabolic process
methionine biosynthetic process
methionine metabolic process
nitrogen compound transport
amino acid salvage
cysteine biosynthetic process from serine
cysteine metabolic process
amino acid transmembrane transport
amino acid transport
membrane
membrane part
integral component of membrane
intrinsic component of membrane
cell cortex part
exocyst
DNA packaging complex
chromatin
nucleosome
protein-DNA complex
transferase activity
ribonucleotide binding
transferase activity, transferring phosphorus-containing groups
protein kinase activity
protein serine/threonine kinase activity
transferase activity, transferring glycosyl groups
transferase activity, transferring hexosyl groups
transferase activity, transferring acyl groups
nucleic acid binding transcription factor activity
serine-type endopeptidase inhibitor activity
transaminase activity
transferase activity, transferring nitrogenous groups
amino acid transmembrane transporter activity
ammonium transmembrane transporter activity
up
down
Biological Process
Cellular Component
Molecular Function

(B)

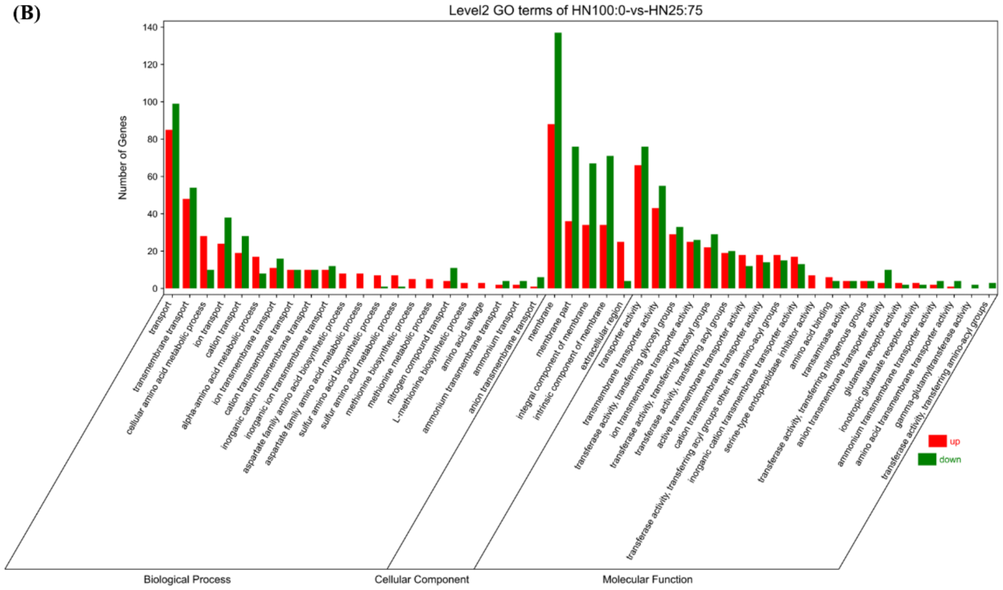
Level2 GO terms of HN100:0-vs-HN25:75
Number of Genes
0
20
40
60
80
100
120
140
transport
transmembrane transport
cellular amino acid metabolic process
ion transport
cation transport
alpha-amino acid metabolic process
ion transmembrane transport
cation transmembrane transport
inorganic cation transmembrane transport
inorganic ion transmembrane transport
aspartate family amino acid biosynthetic process
aspartate family amino acid metabolic process
sulfur amino acid biosynthetic process
sulfur amino acid metabolic process
methionine biosynthetic process
methionine metabolic process
nitrogen compound transport
L-methionine biosynthetic process
amino acid salvage
ammonium transmembrane transport
ammonium transport
anion transmembrane transport
membrane
membrane part
integral component of membrane
intrinsic component of membrane
extracellular region
transporter activity
transmembrane transporter activity
transferase activity, transferring glycosyl groups
ion transmembrane transporter activity
transferase activity, transferring hexosyl groups
transferase activity, transferring acyl groups
active transmembrane transporter activity
cation transmembrane transporter activity
transferase activity, transferring acyl groups other than amino-acyl groups
inorganic cation transmembrane transporter activity
serine-type endopeptidase inhibitor activity
amino acid binding
transaminase activity
transferase activity, transferring nitrogenous groups
anion transmembrane transporter activity
glutamate receptor activity
ionotropic glutamate receptor activity
ammonium transmembrane transporter activity
amino acid transmembrane transporter activity
gamma-glutamyltransferase activity
transferase activity, transferring amino-acyl groups
up
down
Biological Process
Cellular Component
Molecular Function

(C)

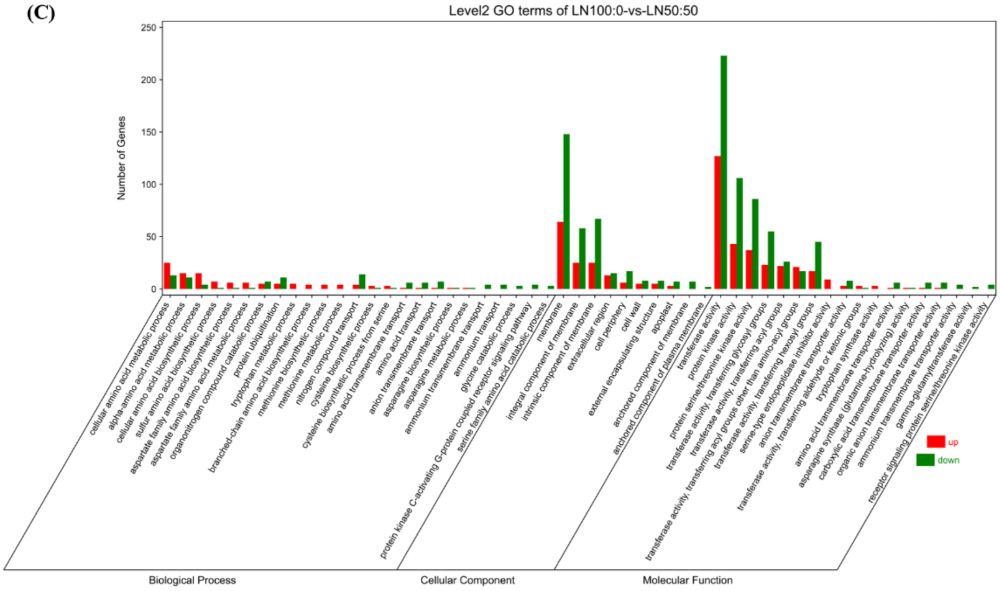
Level2 GO terms of LN100:0-vs-LN50:50
Number of Genes
0
50
100
150
200
250
cellular amino acid metabolic process
alpha-amino acid metabolic process
cellular amino acid biosynthetic process
sulfur amino acid biosynthetic process
aspartate family amino acid biosynthetic process
aspartate family amino acid metabolic process
organonitrogen compound catabolic process
protein ubiquitination
tryptophan metabolic process
branched-chain amino acid biosynthetic process
methionine biosynthetic process
methionine metabolic process
nitrogen compound transport
cysteine biosynthetic process
cysteine biosynthetic process from serine
amino acid transmembrane transport
amino acid transport
anion transmembrane transport
asparagine biosynthetic process
asparagine metabolic process
ammonium transmembrane transport
ammonium transport
glycine catabolic process
protein kinase C-activating G-protein coupled receptor signaling pathway
serine family amino acid catabolic process
membrane
integral component of membrane
intrinsic component of membrane
extracellular region
cell periphery
cell wall
external encapsulating structure
apoplast
anchored component of membrane
anchored component of plasma membrane
transferase activity
protein kinase activity
protein serine/threonine kinase activity
transferase activity, transferring glycosyl groups
transferase activity, transferring acyl groups
transferase activity, transferring acyl groups other than amino-acyl groups
transferase activity, transferring hexosyl groups
serine-type endopeptidase inhibitor activity
anion transmembrane transporter activity
transferase activity, transferring aldehyde or ketonic groups
tryptophan synthase activity
amino acid transmembrane transporter activity
asparagine synthase (glutamine-hydrolyzing) activity
carboxylic acid transmembrane transporter activity
organic anion transmembrane transporter activity
ammonium transmembrane transporter activity
gamma-glutamyltransferase activity
receptor signaling protein serine/threonine kinase activity
up
down
Biological Process
Cellular Component
Molecular Function

(D)

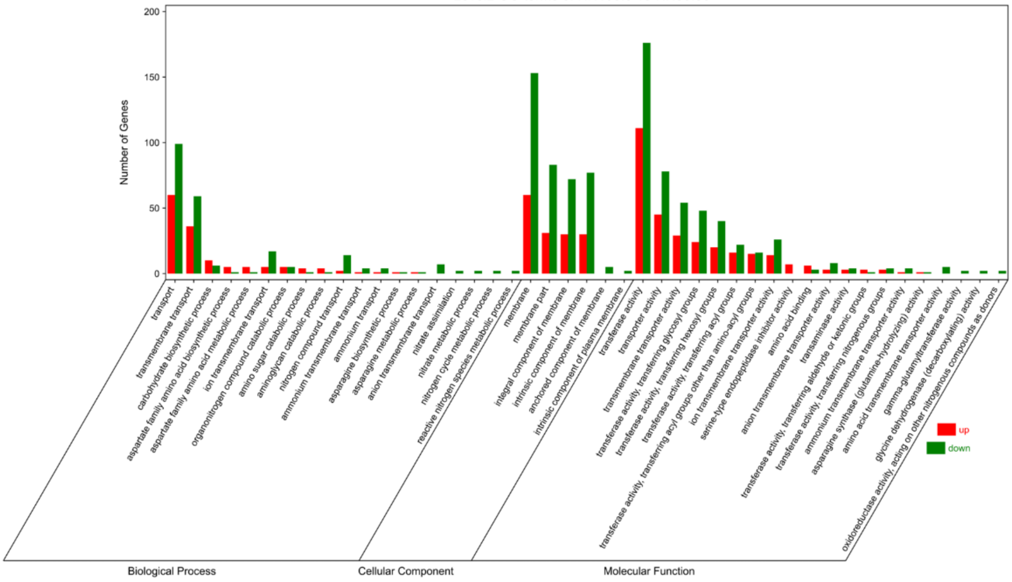
Level2 GO terms of HN100:0-vs-HN50:50
Number of Genes
Biological Process
Cellular Component
Molecular Function
up
down

(E)

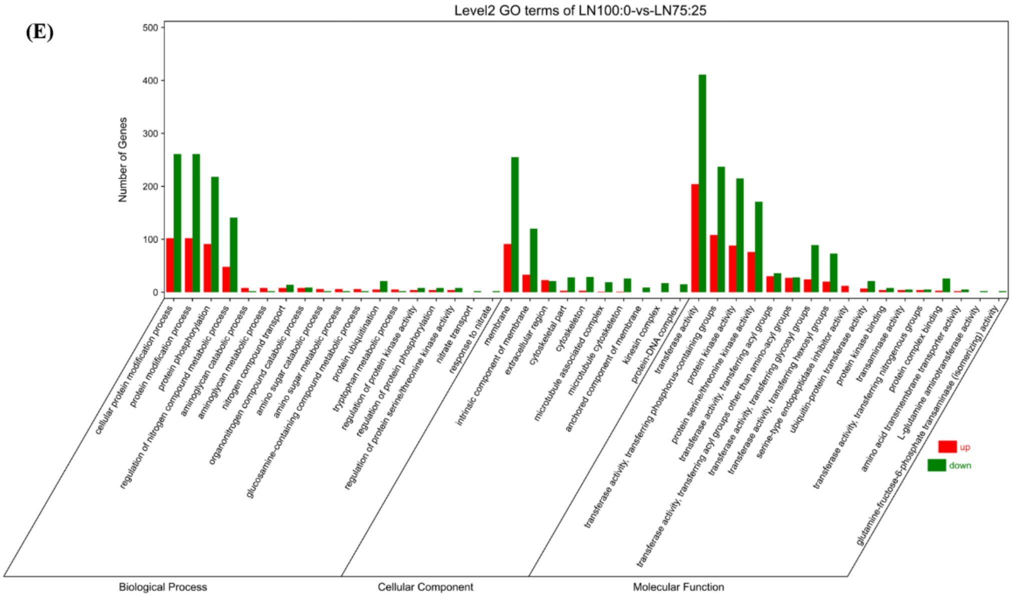
Level2 GO terms of LN100:0-vs-LN75:25
Number of Genes
Biological Process
Cellular Component
Molecular Function
up
down

(F)

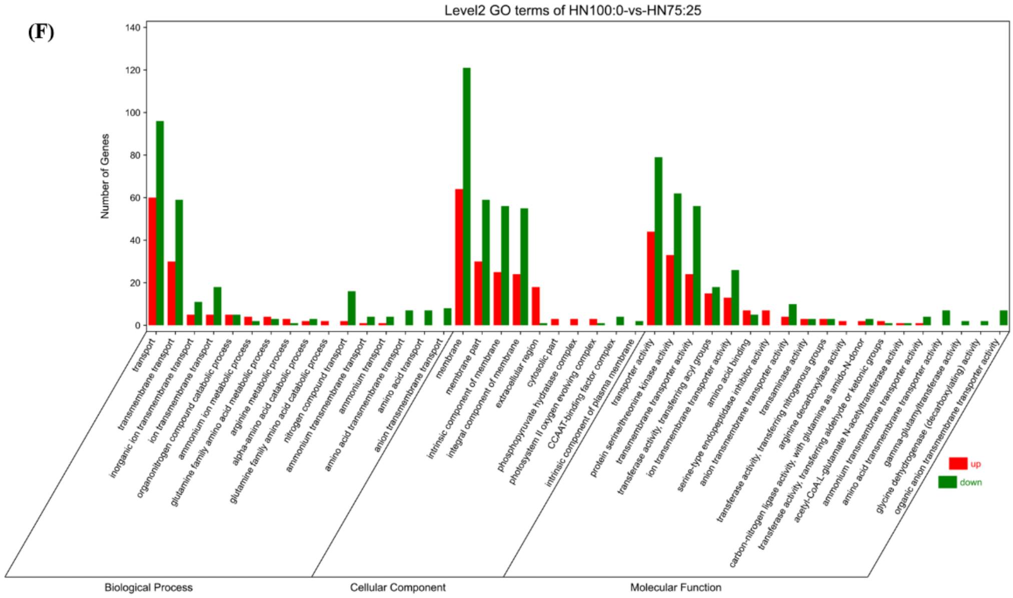
Level2 GO terms of HN100:0-vs-HN75:25
Number of Genes
Biological Process
Cellular Component
Molecular Function
up
down

Figure S1. Significantly enriched GO terms for differentially expressed genes in rice roots under different ammonium–nitrate mixed nutrient conditions. (A) MXZ2 100:0 vs. 25:75 GO enrichment analysis, (B) NJXZ 100:0 vs. 25:75 GO enrichment analysis, (C) MXZ2 100:0 vs. 50:50 GO enrichment analysis, (D) NJXZ 100:0 vs. 50:50 GO enrichment analysis, (E) MXZ2 100:0 vs. 75:25 GO enrichment analysis, (F) NJXZ 100:0 vs. 75:25 GO enrichment analysis.

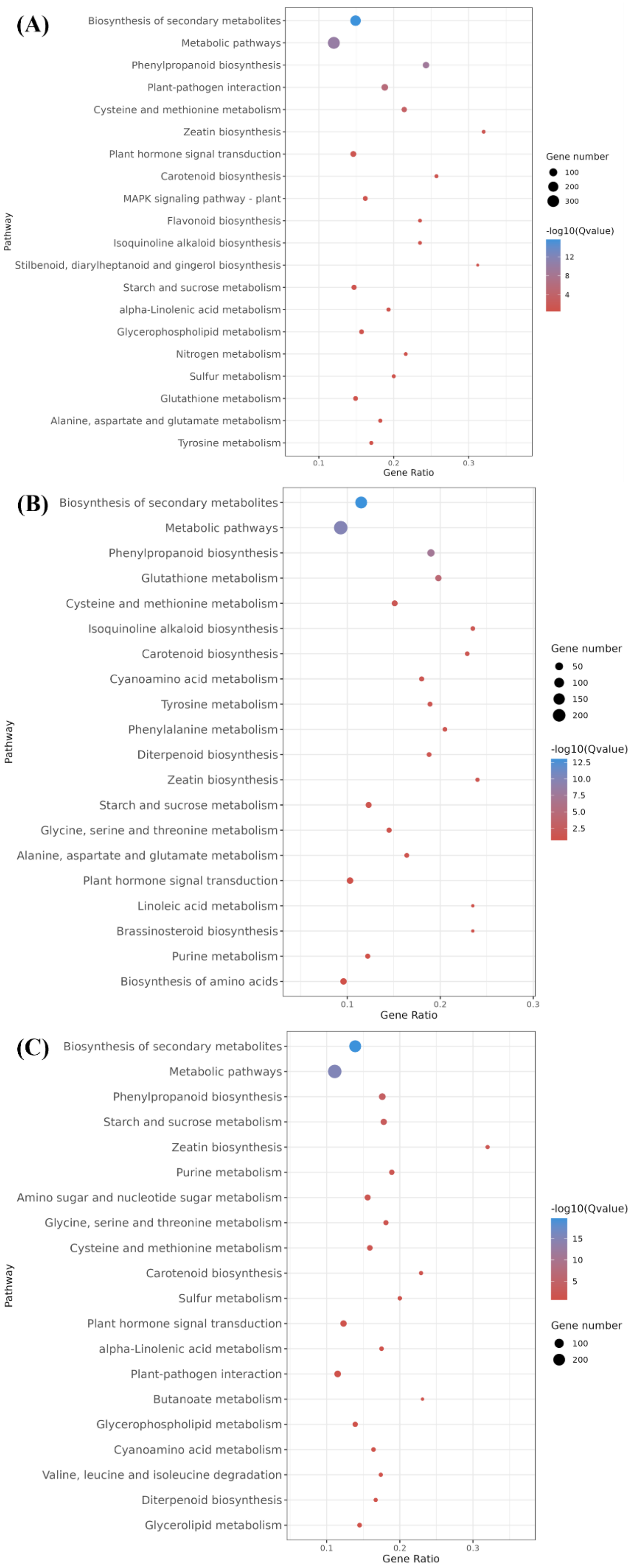
(A)
Biosynthesis of secondary metabolites
Metabolic pathways
Phenylpropanoid biosynthesis
Plant-pathogen interaction
Cysteine and methionine metabolism
Zeatin biosynthesis
Plant hormone signal transduction
Carotenoid biosynthesis
MAPK signaling pathway - plant
Flavonoid biosynthesis
Isoquinoline alkaloid biosynthesis
Stilbenoid, diarylheptanoid and gingerol biosynthesis
Starch and sucrose metabolism
alpha-Linolenic acid metabolism
Glycerophospholipid metabolism
Nitrogen metabolism
Sulfur metabolism
Glutathione metabolism
Alanine, aspartate and glutamate metabolism
Tyrosine metabolism
Pathway
0.1
0.2
0.3
Gene Ratio
Gene number
100
200
300
-log10(Qvalue)
12
8
4
(B)
Biosynthesis of secondary metabolites
Metabolic pathways
Phenylpropanoid biosynthesis
Glutathione metabolism
Cysteine and methionine metabolism
Isoquinoline alkaloid biosynthesis
Carotenoid biosynthesis
Cyanoamino acid metabolism
Tyrosine metabolism
Phenylalanine metabolism
Diterpenoid biosynthesis
Zeatin biosynthesis
Starch and sucrose metabolism
Glycine, serine and threonine metabolism
Alanine, aspartate and glutamate metabolism
Plant hormone signal transduction
Linoleic acid metabolism
Brassinosteroid biosynthesis
Purine metabolism
Biosynthesis of amino acids
Pathway
0.1
0.2
0.3
Gene Ratio
Gene number
50
100
150
200
-log10(Qvalue)
12.5
10.0
7.5
5.0
2.5
(C)
Biosynthesis of secondary metabolites
Metabolic pathways
Phenylpropanoid biosynthesis
Starch and sucrose metabolism
Zeatin biosynthesis
Purine metabolism
Amino sugar and nucleotide sugar metabolism
Glycine, serine and threonine metabolism
Cysteine and methionine metabolism
Carotenoid biosynthesis
Sulfur metabolism
Plant hormone signal transduction
alpha-Linolenic acid metabolism
Plant-pathogen interaction
Butanoate metabolism
Glycerophospholipid metabolism
Cyanoamino acid metabolism
Valine, leucine and isoleucine degradation
Diterpenoid biosynthesis
Glycerolipid metabolism
Pathway
0.1
0.2
0.3
Gene Ratio
-log10(Qvalue)
15
10
5
Gene number
100
200

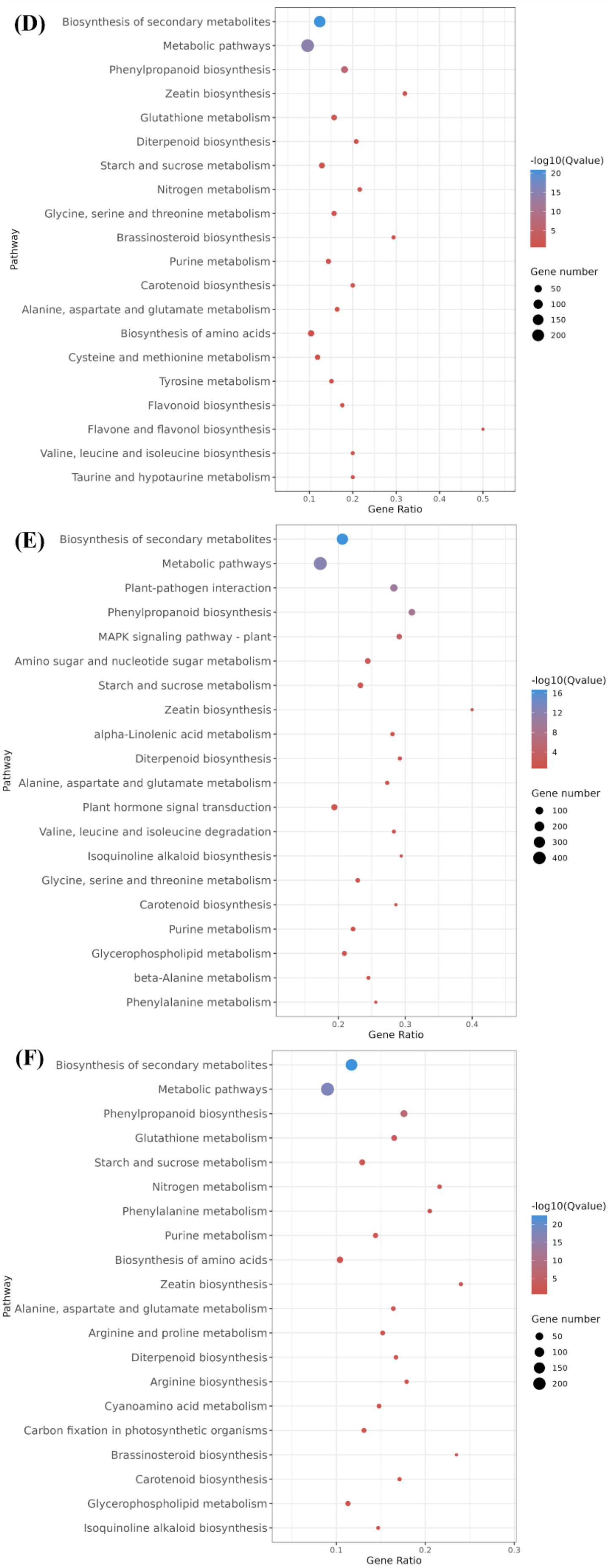

Figure S2. KEGG enrichment bubble plots for pathway enrichment analysis of

rice roots under different ammonium–nitrate mixed nutrient conditions. (A) MXZ2 100:0 vs. 25:75 KEGG enrichment analysis, (B) NJXZ 100:0 vs. 25:75 KEGG enrichment analysis, (C) MXZ2 100:0 vs. 50:50 KEGG enrichment analysis, (D) NJXZ 100:0 vs. 50:50 KEGG enrichment analysis, (E) MXZ2 100:0 vs. 75:25 KEGG enrichment analysis, and (F) NJXZ 100:0 vs. 75:25 KEGG enrichment analysis.

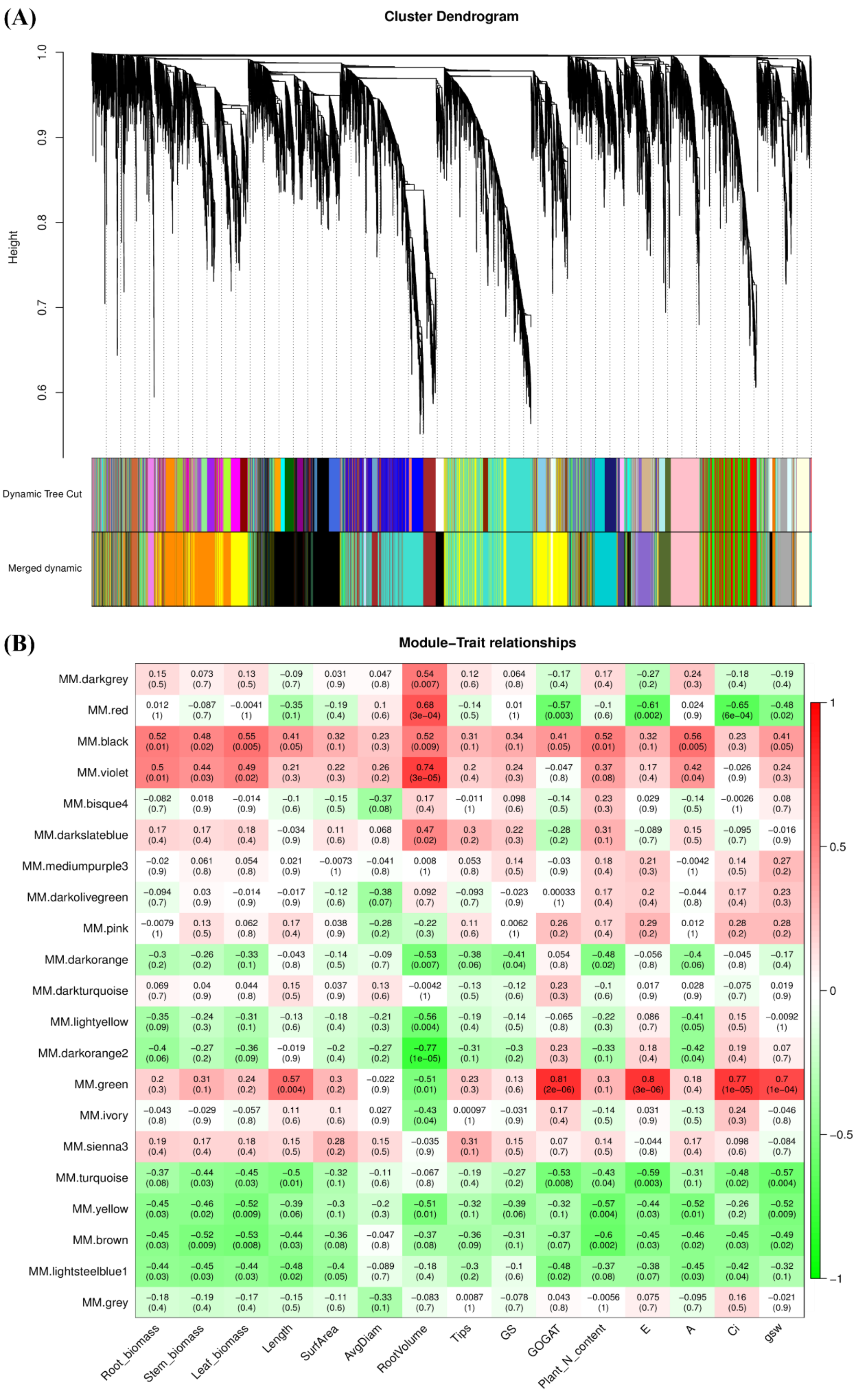
(A)
Cluster Dendrogram
Height
1.0
0.9
0.8
0.7
0.6
Dynamic Tree Cut
Merged dynamic
(B)
Module−Trait relationships
MM.darkgrey 0.15 (0.5) 0.073 (0.7) 0.13 (0.5) −0.09 (0.7) 0.031 (0.9) 0.047 (0.8) 0.54 (0.007) 0.12 (0.6) 0.064 (0.8) −0.17 (0.4) 0.17 (0.4) −0.27 (0.2) 0.24 (0.3) −0.18 (0.4) −0.19 (0.4)
MM.red 0.012 (1) −0.087 (0.7) −0.0041 (1) −0.35 (0.1) −0.19 (0.4) 0.1 (0.6) 0.68 (3e−04) −0.14 (0.5) 0.01 (1) −0.57 (0.003) −0.1 (0.6) −0.61 (0.002) 0.024 (0.9) −0.65 (6e−04) −0.48 (0.02)
MM.black 0.52 (0.01) 0.48 (0.02) 0.55 (0.005) 0.41 (0.05) 0.32 (0.1) 0.23 (0.3) 0.52 (0.009) 0.31 (0.1) 0.34 (0.1) 0.41 (0.05) 0.52 (0.01) 0.32 (0.1) 0.56 (0.005) 0.23 (0.3) 0.41 (0.05)
MM.violet 0.5 (0.01) 0.44 (0.03) 0.49 (0.02) 0.21 (0.3) 0.22 (0.3) 0.26 (0.2) 0.74 (3e−05) 0.2 (0.4) 0.24 (0.3) −0.047 (0.8) 0.37 (0.08) 0.17 (0.4) 0.42 (0.04) −0.026 (0.9) 0.24 (0.3)
MM.bisque4 −0.082 (0.7) 0.018 (0.9) −0.014 (0.9) −0.1 (0.6) −0.15 (0.5) −0.37 (0.08) 0.17 (0.4) −0.011 (1) 0.098 (0.6) −0.14 (0.5) 0.23 (0.3) 0.029 (0.9) −0.14 (0.5) −0.0026 (1) 0.08 (0.7)
MM.darkslateblue 0.17 (0.4) 0.17 (0.4) 0.18 (0.4) −0.034 (0.9) 0.11 (0.6) 0.068 (0.8) 0.47 (0.02) 0.3 (0.2) 0.22 (0.3) −0.28 (0.2) 0.31 (0.1) −0.089 (0.7) 0.15 (0.5) −0.095 (0.7) −0.016 (0.9)
MM.mediumpurple3 −0.02 (0.9) 0.061 (0.8) 0.054 (0.8) 0.021 (0.9) −0.0073 (1) −0.041 (0.8) 0.008 (1) 0.053 (0.8) 0.14 (0.5) −0.03 (0.9) 0.18 (0.4) 0.21 (0.3) −0.0042 (1) 0.14 (0.5) 0.27 (0.2)
MM.darkolivegreen −0.094 (0.7) 0.03 (0.9) −0.014 (0.9) −0.017 (0.9) −0.12 (0.6) −0.38 (0.07) 0.092 (0.7) −0.093 (0.7) −0.023 (0.9) 0.00033 (1) 0.17 (0.4) 0.2 (0.4) −0.044 (0.8) 0.17 (0.4) 0.23 (0.3)
MM.pink −0.0079 (1) 0.13 (0.5) 0.062 (0.8) 0.17 (0.4) 0.038 (0.9) −0.28 (0.2) −0.22 (0.3) 0.11 (0.6) 0.0062 (1) 0.26 (0.2) 0.17 (0.4) 0.29 (0.2) 0.012 (1) 0.28 (0.2) 0.28 (0.2)
MM.darkorange −0.3 (0.2) −0.26 (0.2) −0.33 (0.1) −0.043 (0.8) −0.14 (0.5) −0.09 (0.7) −0.53 (0.007) −0.38 (0.06) −0.41 (0.04) 0.054 (0.8) −0.48 (0.02) −0.056 (0.8) −0.4 (0.06) −0.045 (0.8) −0.17 (0.4)
MM.darkturquoise 0.069 (0.7) 0.04 (0.9) 0.044 (0.8) 0.15 (0.5) 0.037 (0.9) 0.13 (0.6) −0.0042 (1) −0.13 (0.5) −0.12 (0.6) 0.23 (0.3) −0.1 (0.6) 0.017 (0.9) 0.028 (0.9) −0.075 (0.7) 0.019 (0.9)
MM.lightyellow −0.35 (0.09) −0.24 (0.3) −0.31 (0.1) −0.13 (0.6) −0.18 (0.4) −0.21 (0.3) −0.56 (0.004) −0.19 (0.4) −0.14 (0.5) −0.065 (0.8) −0.22 (0.3) 0.086 (0.7) −0.41 (0.05) 0.15 (0.5) −0.0092 (1)
MM.darkorange2 −0.4 (0.06) −0.27 (0.2) −0.36 (0.09) −0.019 (0.9) −0.2 (0.4) −0.27 (0.2) −0.77 (1e−05) −0.31 (0.1) −0.3 (0.2) 0.23 (0.3) −0.33 (0.1) 0.18 (0.4) −0.42 (0.04) 0.19 (0.4) 0.07 (0.7)
MM.green 0.2 (0.3) 0.31 (0.1) 0.24 (0.2) 0.57 (0.004) 0.3 (0.2) −0.022 (0.9) −0.51 (0.01) 0.23 (0.3) 0.13 (0.6) 0.81 (2e−06) 0.3 (0.1) 0.8 (3e−06) 0.18 (0.4) 0.77 (1e−05) 0.7 (1e−04)
MM.ivory −0.043 (0.8) −0.029 (0.9) −0.057 (0.8) 0.11 (0.6) 0.1 (0.6) 0.027 (0.9) −0.43 (0.04) 0.00097 (1) −0.031 (0.9) 0.17 (0.4) −0.14 (0.5) 0.031 (0.9) −0.13 (0.5) 0.24 (0.3) −0.046 (0.8)
MM.sienna3 0.19 (0.4) 0.17 (0.4) 0.18 (0.4) 0.15 (0.5) 0.28 (0.2) 0.15 (0.5) −0.035 (0.9) 0.31 (0.1) 0.15 (0.5) 0.07 (0.7) 0.14 (0.5) −0.044 (0.8) 0.17 (0.4) 0.098 (0.6) −0.084 (0.7)
MM.turquoise −0.37 (0.08) −0.44 (0.03) −0.45 (0.03) −0.5 (0.01) −0.32 (0.1) −0.11 (0.6) −0.067 (0.8) −0.19 (0.4) −0.27 (0.2) −0.53 (0.008) −0.43 (0.04) −0.59 (0.003) −0.31 (0.1) −0.48 (0.02) −0.57 (0.004)
MM.yellow −0.45 (0.03) −0.46 (0.02) −0.52 (0.009) −0.39 (0.06) −0.3 (0.1) −0.2 (0.3) −0.51 (0.01) −0.32 (0.1) −0.39 (0.06) −0.32 (0.1) −0.57 (0.004) −0.44 (0.03) −0.52 (0.01) −0.26 (0.2) −0.52 (0.009)
MM.brown −0.45 (0.03) −0.52 (0.009) −0.53 (0.008) −0.44 (0.03) −0.36 (0.08) −0.047 (0.8) −0.37 (0.08) −0.36 (0.09) −0.31 (0.1) −0.37 (0.07) −0.6 (0.002) −0.45 (0.03) −0.46 (0.02) −0.45 (0.03) −0.49 (0.02)
MM.lightsteelblue1 −0.44 (0.03) −0.45 (0.03) −0.44 (0.03) −0.48 (0.02) −0.4 (0.05) −0.089 (0.7) −0.18 (0.4) −0.3 (0.2) −0.1 (0.6) −0.48 (0.02) −0.37 (0.08) −0.38 (0.07) −0.45 (0.03) −0.42 (0.04) −0.32 (0.1)
MM.grey −0.18 (0.4) −0.19 (0.4) −0.17 (0.4) −0.15 (0.5) −0.11 (0.6) −0.33 (0.1) −0.083 (0.7) 0.0087 (1) −0.078 (0.7) 0.043 (0.8) −0.0056 (1) 0.075 (0.7) −0.095 (0.7) 0.16 (0.5) −0.021 (0.9)
Root_biomass
Stem_biomass
Leaf_biomass
Length
SurfArea
AvgDiam
RootVolume
Tips
GS
GOGAT
Plant_N_content
E
A
Ci
gsw
1
0.5
0
−0.5
−1

Black module

(C)

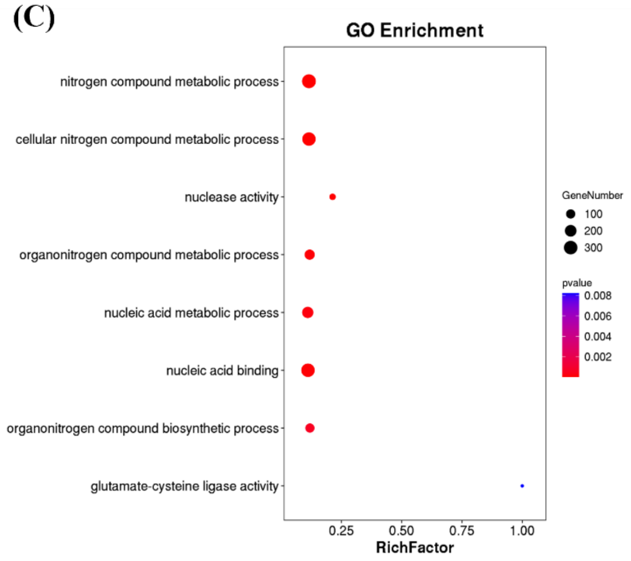

Green module

(D)

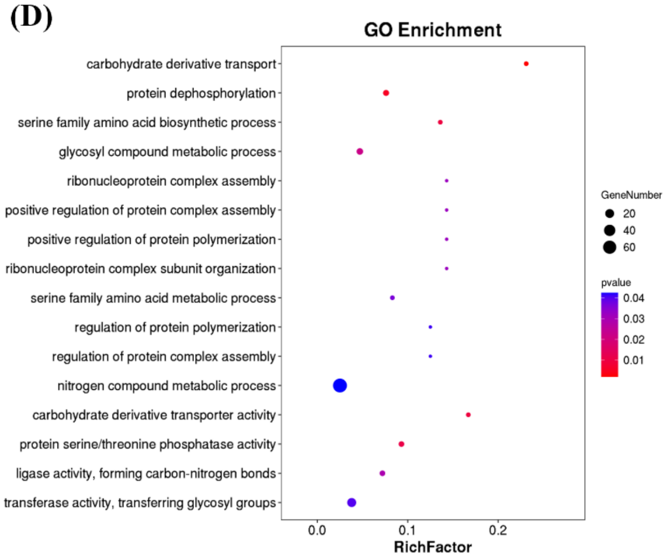

Violet module

(E)

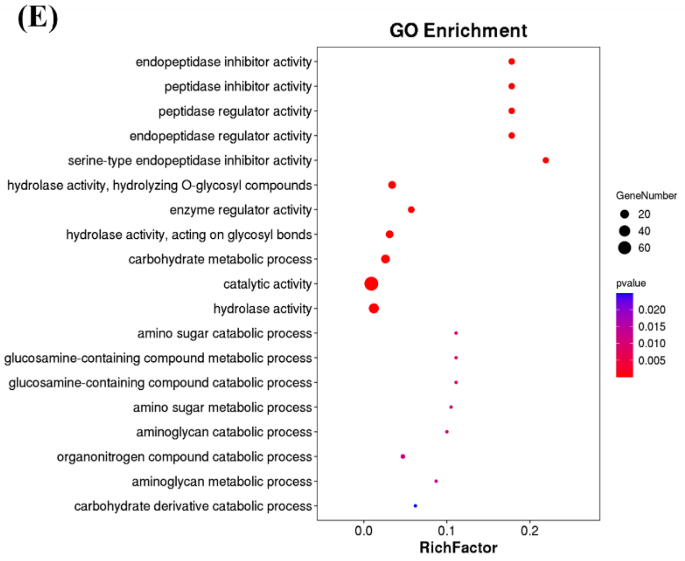

Figure S3. Weighted gene coexpression network analysis in rice. The figure shows the (A) division of gene modules, (B) correlation analysis of genes with phenotypic data in each module, (C) enrichment analysis of key genes in the black module, (D) enrichment analysis of key genes in the green module, (E) enrichment analysis of key genes in the purple module
